# Supplementary material for: A leap in quantum efficiency through light harvesting in photoreceptor UVR8
Source: Nat Commun. 2020 Aug 28;11:4316. doi: 10.1038/s41467-020-17838-6 (PMC7455749; doi:10.1038/s41467-020-17838-6)
Supplement: Supplementary file 1 — Supplementary Information [file 41467_2020_17838_MOESM1_ESM.pdf]

# Supplementary Information

## A leap in quantum efficiency through light harvesting in photoreceptor UVR8

Li et al.

# Supplementary Methods

## Model simulation of picosecond resolved TCSPC transients

*1. Simulation of Excited State Population Evolution of Three W Groups*

The time evolution of excited state populations of UVR8 tryptophan residues were numerically simulated (from 0 to 30 ns, 4 ps interval) based on kinetic models. Parameters are shown in Supplementary Tables 2, 15 and 16. The RET time constants from QM/MM calculations in Supplementary Table 3 and Fig. 3d were also needed.

Since $W_d$ has two lifetimes, for each $W_d$, the excited state population was treated as the sum of two subpopulations with the same energy-transfer rate but with distinct lifetimes (Supplementary equation 1), 0.5 ns for $W_d1$ and 2.7 ns for $W_d2$. Knowing all lifetimes, excitation energy-transfer total rates (see equation 17) and absorbance values (Fig. 1e and Supplementary Table 5), the differential equations and corresponding boundary conditions describing the population evolution of a distal tryptophan Wm (m=39, 92, 144, 196, 300 or 352) are shown as below:

$$[Wm]_t = [Wm_1]_t + [Wm_2]_t \tag{1}$$

$$\frac{d[Wm_1]_t}{dt} = -(\tau_{total,Wm}{}^{-1} + \tau_1{}^{-1})[Wm_1]_t \qquad [Wm_1]_{t=0} = A_d(\lambda)(1-R_{D2}) \tag{2}$$

$$\frac{d[Wm_2]_t}{dt} = -(\tau_{total,Wm}{}^{-1} + \tau_2{}^{-1})[Wm_2]_t \qquad [Wm_2]_{t=0} = A_d(\lambda)R_{D2} \tag{3}$$

Supplementary equations 2 and 3 define the decay dynamics of the 0.5 ns and 2.7 ns components, respectively, and they are single exponential decays. In the equations, $\tau_{total,Wm}$ is the theoretical total RET time constant (Fig. 2k) calculated using equation 17 for Wm (m=39, 92, 144, 196, 300 or 352). $\tau_1$ and $\tau_2$ are the two lifetimes of $W_d$, which are 0.5 ns and 2.7 ns, respectively. $R_{D2}$ is the fraction of amplitude for the 2.7 ns lifetime, which is 0.71 as determined by femtosecond fluorescence up-conversion (Fig. 2a). The initial populations are based on normalized $W_d$ absorption spectrum ($A_d(\lambda)$ values, Supplementary Table 5, Fig. 1e) at the excitation wavelength $\lambda$ ($\lambda$=290 nm or 310 nm). The decay curves of all $W_d$ were then added together to obtain the total dynamics of distal tryptophan group:

$$[W_d]_t = [W39]_t + [W92]_t + [W144]_t + [W196]_t + [W300]_t + [W352]_t \tag{4}$$

For each peripheral tryptophan residues Wi (i=198, 250 or 302), depending on the lifetime and energy transfer rates, the total excited population was divided into 4 subpopulations: (1) short lifetime (1-2 ns) and fast energy transfer to $4W_c$, (2) long lifetime (6-8 ns) and fast energy transfer to $4W_c$, (3) short lifetime and slow energy transfer to $4W_c$, (4) long lifetime and slow energy transfer to $4W_c$.

$$[Wi]_t = [Wi_1]_t + [Wi_2]_t + [Wi_3]_t + [Wi_4]_t \tag{5}$$

Each subpopulation has a distinct differential equation and boundary conditions for numerical simulation:

$$\frac{d[Wi_1]_t}{dt} = -(\tau_{total1,Wi}{}^{-1} + \tau_{1,Wi}{}^{-1})[Wi_1]_t + (1-R_{slow,Wi})(1-R_{2,Wi})\sum_m^{W_d}(\tau_{RET,WmWi}{}^{-1} + \tau_{RET,WmWi(b)}{}^{-1})[Wm]_t$$

$$[Wi_1]_{t=0} = A_p(\lambda)(1-R_{slow,Wi})(1-R_{2,Wi}) \tag{6}$$

$$\frac{d[Wi_2]_t}{dt} = -(\tau_{total1,Wi}{}^{-1} + \tau_{2,Wi}{}^{-1})[Wi_2]_t + (1 - R_{slow,Wi})R_{2,Wi}\sum_m^{W_d}(\tau_{RET,WmWi}{}^{-1} + \tau_{RET,WmWi(b)}{}^{-1})[Wm]_t$$

$$[Wi_2]_{t=0} = A_p(\lambda)(1 - R_{slow,Wi})R_{2,Wi} \tag{7}$$

$$\frac{d[Wi_3]_t}{dt} = -(\tau_{total2,Wi}{}^{-1} + \tau_{1,Wi}{}^{-1})[Wi_3]_t + R_{slow,Wi}(1 - R_{2,Wi})\sum_m^{W_d}(\tau_{RET,WmWi}{}^{-1} + \tau_{RET,WmWi(b)}{}^{-1})[Wm]_t$$

$$[Wi_3]_{t=0} = A_p(\lambda)R_{slow,Wi}(1 - R_{2,Wi}) \tag{8}$$

$$\frac{d[Wi_4]_t}{dt} = -(\tau_{total2,Wi}{}^{-1} + \tau_{2,Wi}{}^{-1})[Wi_4]_t + R_{slow,Wi}R_{2,Wi}\sum_m^{W_d}(\tau_{RET,WmWi}{}^{-1} + \tau_{RET,WmWi(b)}{}^{-1})[Wm]_t$$

$$[Wi_4]_{t=0} = A_p(\lambda)R_{slow,Wi}R_{2,Wi} \tag{9}$$

Supplementary equations 6, 7, 8 and 9 describe the temporal evolution of the 4 excited state subpopulations, respectively. On the right side of each equation, the first term gives the decay due to lifetime decay and energy transfer to $W_c$; the second term defines the population increase kinetics due to energy transfer from $W_d$. In the above equations, $\tau_{total1,Wi}$ and $\tau_{total2,Wi}$ are the fast and slow QM/MM total RET time constant from peripheral tryptophan Wi (i=198, 250 or 302) to the 4 pyramid center tryptophan residues (shown in Fig. 3d). $\tau_{1,Wi}$ and $\tau_{2,Wi}$ are the two lifetimes of Wi. $\tau_{RET,WmWi}$ is the RET time constant from one distal tryptophan Wm to the peripheral tryptophan Wi and $\tau_{RET,WmWi(b)}$ is the RET time constant from the same $W_d$ to the $W_p$ on the other subunit (Supplementary Table 3). $[Wm]_t$ is the population evolution of excited distal tryptophan m (m=39, 92, 144, 196, 300 or 352) simulated as above mentioned. $R_{2,Wi}$ is the fraction of amplitude for the longer lifetime (6-8 ns) and $R_{slow,Wi}$ stands for the population ratio of $W_p$ with slow transfer energy to $W_c$ (shown in Fig. 3d). $A_p(\lambda)$ is the value of the normalized $W_p$ absorption spectrum (shown in Fig. 1e and Supplementary Table 5) at the excitation wavelength $\lambda$ ($\lambda$=290 nm or 310 nm), which determines initial population of $W_p$. For $W_p$ that were knocked out in the mutant, the $A_p(\lambda)$ value was set to 0. For each mutant, the simulated transients for peripheral tryptophan residues present in the mutant were added together to give the total dynamics of peripheral tryptophan group:

$$[W_p]_t = \sum_i [Wi]_t \tag{10}$$

Our model treated the 4 pyramid center tryptophan residues ($4W_c$) as a whole. Since 2 decay time constants were observed (0.08 ns and 1.4 ns) for the pyramid center, we divide $W_c$ into two subpopulations:

$$[W_c]_t = [W_{c1}]_t + [W_{c2}]_t \tag{11}$$

$$\frac{d[W_{c1}]_t}{dt} = -\tau_{c1}{}^{-1}[W_{c1}]_t + R_{c1}\sum_m^{W_d}\sum_n^{W_c}(\tau_{RET,WmWn}{}^{-1} + \tau_{RET,WmWn(b)}{}^{-1})[Wm]_t + R_{c1}\sum_i^{W_p}\tau_{total1,Wi}{}^{-1}([Wi_1]_t + [Wi_2]_t)$$

$$+ R_{c1}\sum_i^{W_p}\tau_{total2,Wi}{}^{-1}([Wi_3]_t + [Wi_4]_t)$$

$$[W_{c1}]_{t=0} = 4A_c(\lambda)R_{c1} \tag{12}$$

$$\frac{d[W_{c2}]_t}{dt} = -\tau_{c2}^{-1}[W_{c2}]_t + (1-R_{c1})\sum_m^{W_d}\sum_n^{W_c}(\tau_{RET,WmWn}{}^{-1} + \tau_{RET,WmWn(b)}{}^{-1})[Wm]_t$$

$$+(1-R_{c1})\sum_i^{W_p}\tau_{total1,Wi}{}^{-1}([Wi_1]_t + [Wi_2]_t) + (1-R_{c1})\sum_i^{W_p}\tau_{total2,Wi}{}^{-1}([Wi_3]_t + [Wi_4]_t)$$

$$[W_{c2}]_{t=0} = 4A_c(\lambda)(1-R_{c1}) \quad (13)$$

In Supplementary equations 12 and 13, $\tau_{c1}$ and $\tau_{c2}$ are the two fluorescence decay times of the pyramid center, which are 0.08 ns and 1.4 ns, respectively. $\tau_{RET,WmWn}$ and $\tau_{RET,WmWn(b)}$ are the RET time constants from one distal tryptophan Wm (m=39, 92, 144, 196, 300 or 352) to the pyramid center tryptophan Wn on the same and on the other subunit (Supplementary Table 3), respectively. $[Wm]_t$ is the kinetics of excited distal tryptophan m (m=39, 92, 144, 196, 300 or 352) simulated as above mentioned. $\tau_{total1,Wi}$ and $\tau_{total2,Wi}$ are the fast and slow QM/MM total RET time constant from peripheral tryptophan Wi (i=198, 250 or 302) to the 4 pyramid center tryptophan residues (Fig. 3d). $[Wi_1]_t$, $[Wi_2]_t$, $[Wi_3]_t$ and $[Wi_4]_t$ are the temporal evolutions of the four subpopulations (as above mentioned) of peripheral tryptophan Wi. $R_{c1}$ is the amplitude ratio of the 80 ps component, which is 0.75. $A_c(\lambda)$ is the value of the normalized $W_c$ absorption spectrum (Supplementary Table 5, shown in Fig. 1e) at the excitation wavelength $\lambda$ ($\lambda$=290 nm or 310 nm). Simulations were conducted for W198F, W250F and W198/250F at 310-nm excitation; and WT at 310-nm and 290-nm excitation. Simulated tryptophan excited state temporal evaluation of WT (290 nm excitation) is shown in Supplementary Fig. 16 as an example.

*2. Simulation of Fluorescence Transients and Contributions from Three Tryptophan Groups*

The total simulated signal (Simul.) was given by the sum of contributions from $W_d$, $W_p$ and $W_c$:

$$Simul.(t) = S(W_d) + S(W_p) + S(W_c) \quad (14)$$

Three groups have different emission rates and thus have different weights in the total signal. The instrument response of TCSPC also needs to be considered. Therefore, the contributions were calculated using the following equations:

$$S(W_d) = A \bullet FL_d(\lambda)\int_{-\infty}^{+\infty} IRF(\tau)[W_d]_{t-\tau}d\tau \quad (15)$$

$$S(W_p) = A \bullet FL_p(\lambda)\int_{-\infty}^{+\infty} IRF(\tau)[W_p]_{t-\tau}d\tau \quad (16)$$

$$S(W_c) = A \bullet FL_c(\lambda)\int_{-\infty}^{+\infty} IRF(\tau)[W_c]_{t-\tau}d\tau \quad (17)$$

where $[W_d]$, $[W_p]$ and $[W_c]$ are the kinetics of excited distal, peripheral and pyramid center tryptophan chromophores as defined in Supplementary equations 4, 10 and 11, respectively. IRF is the instrument response function as recorded using buffer scattering (Supplementary Fig. 15a). $FL_d$, $FL_p$ and $FL_c$ are the fluorescence intensity of $W_d$, $W_p$ and $W_c$ per unit population per unit time, respectively, giving different signal weights to 3 W groups. $FL_d$, $FL_p$ and $FL_c$ have the same spectra shapes as fluorescence spectra shown in Fig. 1e, but with different absolute intensities

(Supplementary Table 19), which were determined by fluorescence QY and lifetimes of 3 W groups (Supplementary Fig. 15). For comparison with experimental data, a common normalizing factor (A) was applied to Supplementary equations 15, 16 and 17 to give a normalized total simulation curve without affecting the dynamics.

For different mutants, picosecond resolved TCSPC data at multiple fluorescence wavelengths were simulated using our model (Supplementary Figs. 9-13). Given the fact that the steady-state emission is the sum of time integrals of time-resolved fluorescence from 3 W groups (Supplementary equation 18), with the steady-state emission spectra of the mutant and simulation data at various fluorescence wavelengths, the spectra of $W_d$, $W_p$ and $W_c$ can be determined by decomposing the steady-state emission.

$$I(\lambda) = I_d(\lambda) + I_p(\lambda) + I_c(\lambda) \tag{18}$$

$$I_d(\lambda) = I(\lambda)\frac{\int_0^{+\infty} FL_d(\lambda)[W_d]_t\,dt}{\int_0^{+\infty} FL_d(\lambda)[W_d]_t\,dt + \int_0^{+\infty} FL_p(\lambda)[W_p]_t\,dt + \int_0^{+\infty} FL_c(\lambda)[W_c]_t\,dt} \tag{19}$$

$$I_p(\lambda) = I(\lambda)\frac{\int_0^{+\infty} FL_p(\lambda)[W_p]_t\,dt}{\int_0^{+\infty} FL_d(\lambda)[W_d]_t\,dt + \int_0^{+\infty} FL_p(\lambda)[W_p]_t\,dt + \int_0^{+\infty} FL_c(\lambda)[W_c]_t\,dt} \tag{20}$$

$$I_c(\lambda) = I(\lambda)\frac{\int_0^{+\infty} FL_c(\lambda)[W_c]_t\,dt}{\int_0^{+\infty} FL_d(\lambda)[W_d]_t\,dt + \int_0^{+\infty} FL_p(\lambda)[W_p]_t\,dt + \int_0^{+\infty} FL_c(\lambda)[W_c]_t\,dt} \tag{21}$$

where $I(\lambda)$ is the total steady-state emission spectrum. The shapes of resulted $I_d$, $I_p$ and $I_c$ (Fig. 3l-o colored symbols) agree well with directly measured emission spectra of $W_d$, $W_p$ and $W_c$ groups, which further validates our energy transfer model.

**Overall RET efficiency of UVR8**

Details about FRET efficiency calculations can be found elsewhere[1]. Briefly, RET efficiency (E) can be calculated with donor lifetimes with and without acceptors:

$$E = 1 - \frac{\tau_{DA}}{\tau_D} \tag{22}$$

where $\tau_{DA}$ and $\tau_D$ are donor fluorescence decay times with and without the acceptors respectively. In our study, $\tau_D$ was experimentally measured when the acceptors were removed by site-directed mutagenesis. $\tau_{DA}$ was calculated using $\tau_D$ and theoretically calculated total RET rates $\tau_{total}$ (defined in equation 17):

$$\tau_{DA} = \frac{1}{\tau_D^{-1} + \tau_{total}^{-1}} \tag{23}$$

Equations 21 and 22 were readily obtained with Supplementary equations 22 and 23.

For WT, the energy transfer efficiency from $W_d$ directly to the pyramid center ($4W_c$) was

calculated for each $W_d$.

$$E_{direct,Wm4W_c} = E_{Wm}\frac{R_{Wm}}{R_{Wm}+1} \tag{24}$$

$E_{Wm}$ is the RET efficiency for Wm (m=39, 92, 144, 196, 300 or 352) in WT as calculated with equation 20. $R_{Wm}$ is the RET branching ratio of direct transfer for Wm shown in Supplementary Table 3.

The energy transfer efficiency from each $W_d$ to certain $W_p$ was calculated as follows:

$$E_{WmWi} = E_{Wm}\frac{\tau_{RET,WmWi}{}^{-1} + \tau_{RET,WmWi(b)}{}^{-1}}{\tau_{total,Wm}{}^{-1}} \tag{25}$$

$\tau_{total,Wm}$ is the QM/MM total RET time constant defined in equation 14 (shown in Fig. 2k) for Wm (m=39, 92, 144, 196, 300 or 352). $\tau_{RET,WmWi}$ and $\tau_{RET,WmWi(b)}$ are the RET time constants for D-A pairs Wm-Wi and Wm-Wib (i=198, 250 or 302) respectively (data shown in Supplementary Table 3).

The energy transfer efficiency from each $W_d$ to the pyramid center via two-step transfer through $W_p$ was calculated using the following equation:

$$E_{twostep,Wm4W_c} = E_{W198}E_{WmW198} + E_{W250}E_{WmW250} + E_{W302}E_{WmW302} \tag{26}$$

$E_{W198}$, $E_{W250}$ and $E_{W302}$ are the energy transfer efficiencies to the pyramid center given by equation 24. $E_{WmW198}$, $E_{WmW250}$ and $E_{WmW302}$ are the energy transfer efficiencies from Wm (m=39, 92, 144, 196, 300 or 352) to W198, W250 and W302 respectively, calculated using Supplementary equation 25.

Total efficiencies for $W_d$ energy transfer to the pyramid center is the sum of efficiencies of the direct and indirect transfer.

$$E_{Wm4W_c} = E_{direct,Wm4W_c} + E_{twostep,Wm4W_c} \tag{27}$$

All results are shown in Supplementary Table 4.

Finally, the fraction of absorbed photons ($\lambda_{ex}$=290 nm) that is ultimately utilized to excite the pyramid center in UVR8 WT was given by the following equation:

$$E_{290nm} = \frac{A_d(290nm)\sum_{m}^{W_d} E_{Wm4W_c} + A_p(290nm)\sum_{i}^{W_p} E_{Wi} + 4A_c(290nm)}{6A_d(290nm)+3A_p(290nm)+4A_c(290nm)} \tag{28}$$

By plugging in the efficiency values in Supplementary Table 4 and absorbance values in Supplementary Table 5, the light harvesting efficiency in UVR8 was calculated as follows:

$$E_{290nm} = \frac{0.668\times(0.34+0.37+0.49+0.35+0.52+0.51)+0.846\times(0.63+0.94+0.96)+4\times0.888}{6\times0.668+3\times0.846+4\times0.888} = 0.73 \tag{29}$$

meaning that for every 100 photons absorbed by UVR8 WT, about 73 of them finally reach the pyramid center. If all energy transfer efficiencies (E) are set to zero, Supplementary equation 28 gives the probability of direct excitation of the pyramid center, which is 0.35, indicating the energy transfer network increases the light perception efficiency from 35% to 73%.

The total efficiency of the 80 ps photoreaction channel ($\lambda_{ex}$=290 nm) is:

$$E_Q = R_{c1}E_{290nm} = 0.75\times0.73 = 0.55 \tag{30}$$

With $R_{c1}$=0.75, $E_Q$ was calculated to be 0.55, suggesting that for every 100 photons absorbed by UVR8 WT, about 55 of them were quenched via the 80 ps channel.

**Supplementary Figures**

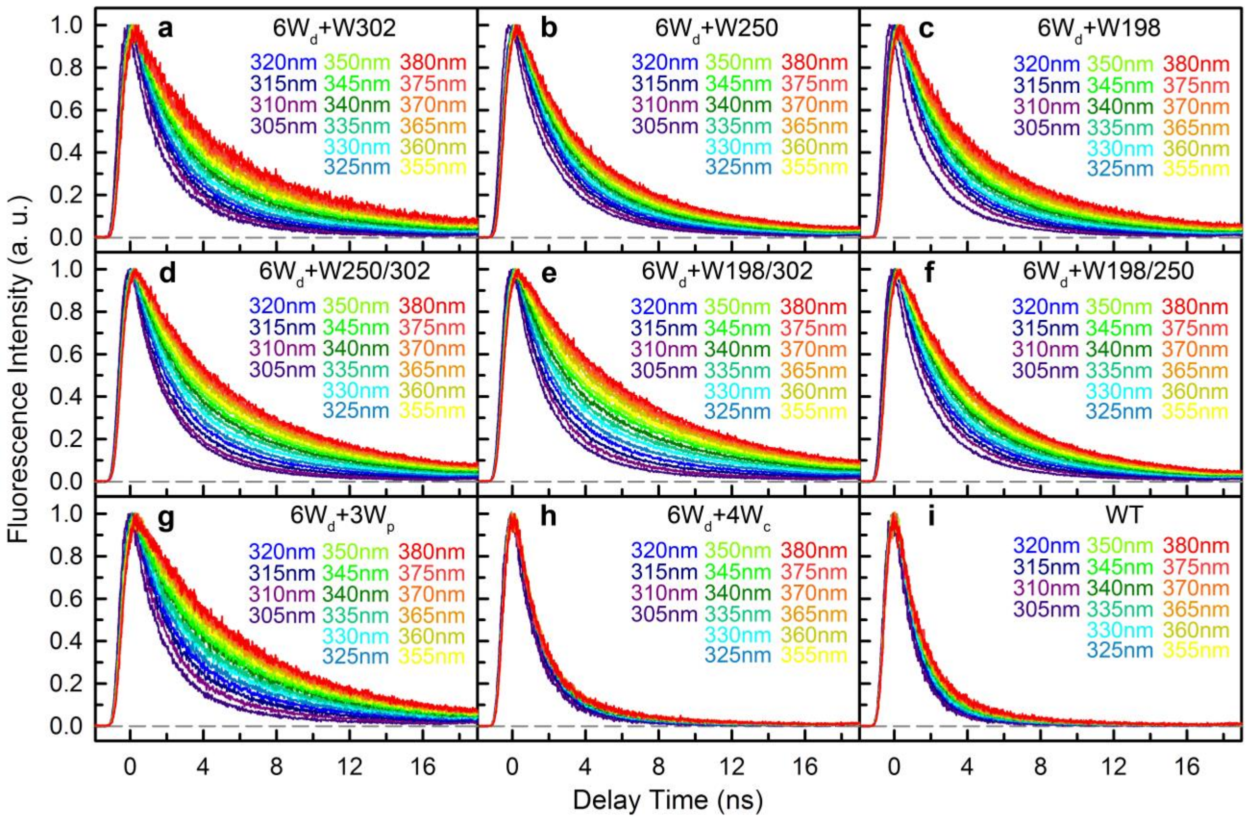


**Supplementary Figure 1 | Fluorescence decay transients ($\lambda_{ex}$=290 nm) from 305 nm to 380 nm for UVR8 WT and 8 mutants measured with sub-nanosecond-resolved time correlated single photon counting (TCSPC).** **a**, 4W$_c$/198/250F (6W$_d$+W302). **b**, 4W$_c$/198/302F (6W$_d$+W250). **c**, 4W$_c$/250/302F (6W$_d$+W198). **d**, 4W$_c$/198F (6W$_d$+W250/302). **e**, 4W$_c$/250F (6W$_d$+W198/302). **f**, 4W$_c$/302F (6W$_d$+W198/250). **g**, 6W$_d$+3W$_p$. **h**, 6W$_d$+4W$_c$. **i**, WT.

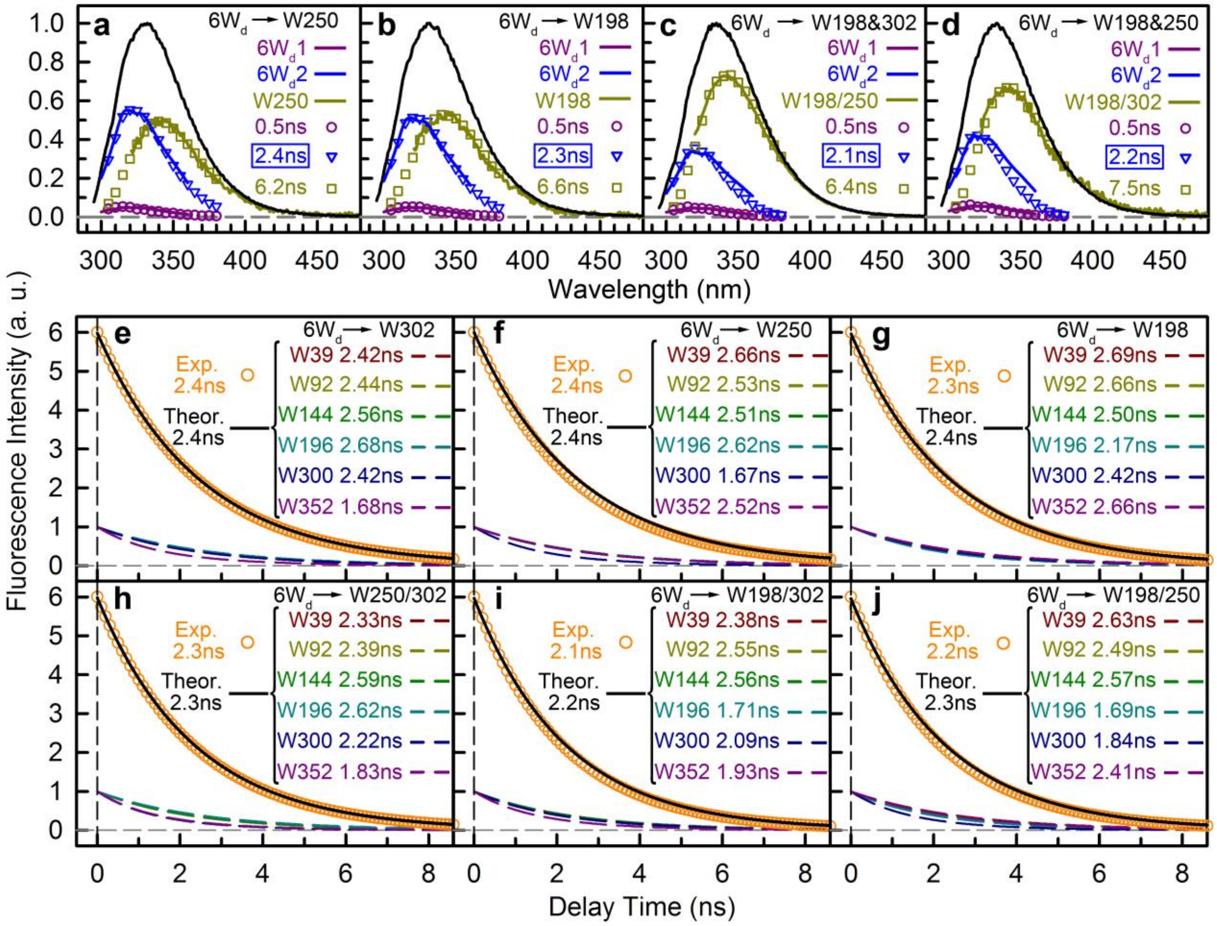


**Supplementary Figure 2 | Excitation energy transfer from $W_d$ to $W_P$ and $W_c$. a-d**, Time scales from a global fitting and lifetime-associated spectra of $6W_d$+W250 (**a**), $6W_d$+W198 (**b**), $6W_d$+W198 & W302 (**c**) and $6W_d$+W198 & W250 (**d**). For each panel, the black solid lines are the steady-state emission spectra of the protein samples. The lifetime-associated spectra are shown in various symbols and the directly measured spectra are shown in lines. **e-j**, Model simulations demonstrate that the total fluorescence decay dynamics of the $6W_d$ in UVR8 can be described with single exponential decays in the following UVR8 protein samples: $4W_c$/198/250F (**e**), $4W_c$/198/302F (**f**), $4W_c$/250/302F (**g**), $4W_c$/198F (**h**), $4W_c$/250F (**i**), $4W_c$/302F (**j**). In each panel, the 6 dashed lines correspond to the simulated single exponential decays of the original 2.7-ns component based on experimental $\tau_2$ (2.7 ns) and theoretical total RET times ($\tau_{total}$) for W39 (dark red), W92 (dark yellow), W144 (green), W196 (dark cyan), W300 (dark blue) and W352 (dark purple) respectively. The simulated total decay dynamics of the $6W_d$ (black solid lines, sum of the 6 dashed lines) can be fitted with a single decay time constant (shown in black), which agrees well with experimental dynamics (yellow circles), indicating that FRET theory provides accurate description of the energy transfer processes from $W_d$ in UVR8.

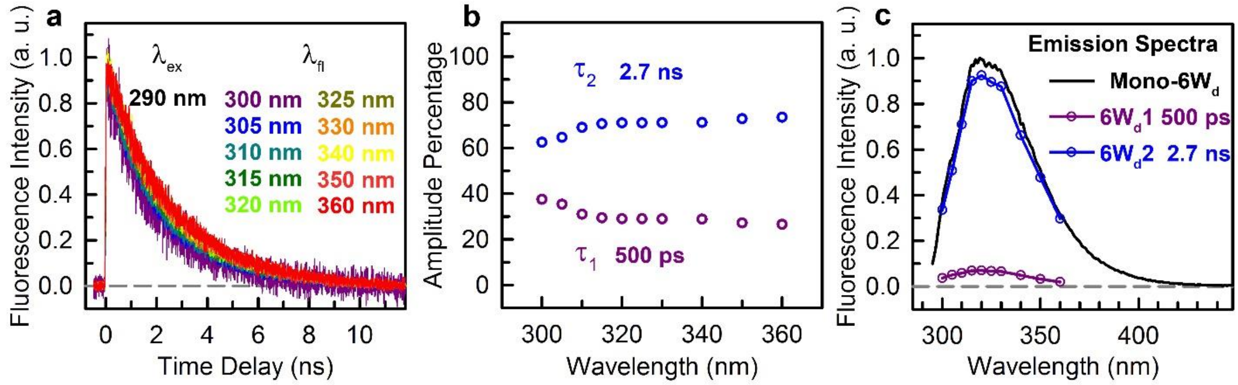


**Supplementary Figure 3 | Lifetime-associated spectra of $W_d$. a**, Fluorescence transients (290 nm excitation) of mono-6$W_d$ measured by picosecond-resolved TCSPC. For each plot, the corresponding fluorescence wavelength is labeled with the same color. **b**, Amplitude percentages of 0.5 ns and 2.7 ns lifetimes at different fluorescence wavelengths as determined by biexponential fitting of the fluorescence transients in **a**. **c**, Lifetime-associated spectra of 0.5 ns (6$W_d$1) and 2.7 ns (6$W_d$2) time constants.

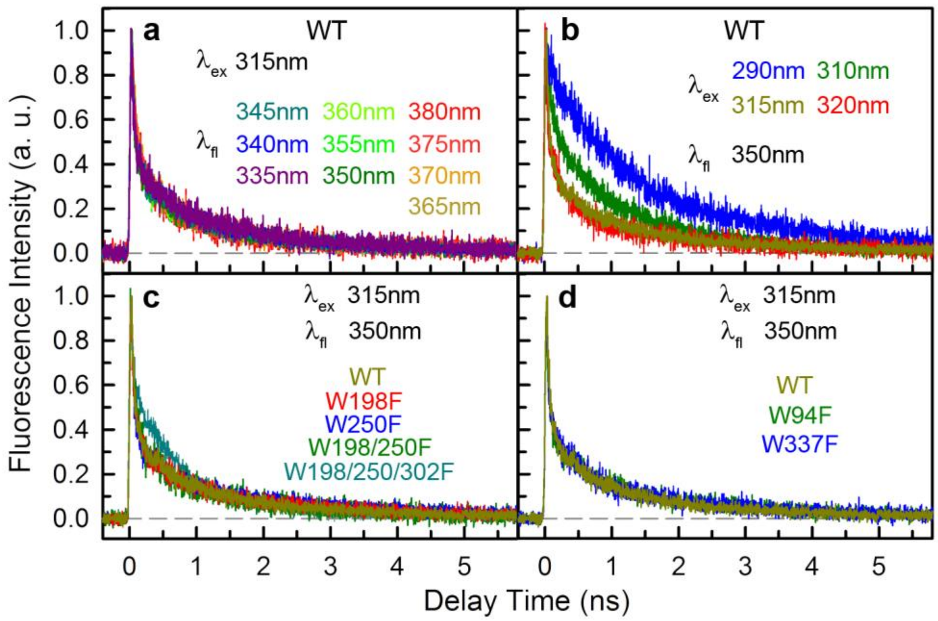


**Supplementary Figure 4 | Fluorescence decay transients measured with picosecond resolved TCSPC.** **a**, Decay transients at various fluorescence wavelengths for WT *(*$\lambda_{ex}$=315 nm). All the transients are similar. **b**, Decay transients at 350 nm with various excitation wavelengths *(*$\lambda_{ex}$=290 nm, 310 nm, 315 nm and 320 nm) for WT. Fluorescence decay signals with 315 nm and 320 nm excitation wavelengths gives pure pyramid center ($4W_c$) dynamics. In addition to $4W_c$ dynamics, 310 nm excitation transients also have contributions from $3W_p$; 290 nm excitation transients contain dynamics of all three groups. **c**, Fluorescence dynamics of the pyramid center measured by 315 nm excitation, for WT (dark yellow), W198F (red), W250F (blue), W198/250F (green) and W198/250/302F ($6W_d$+$4W_c$, cyan). All transients are similar except that for W198/250/302F ($6W_d$+$4W_c$), indicating that mutation of W302 affects the pyramid center fluorescence dynamics and gives an extra 5.4-ns slow decay constant. **d**, Fluorescence dynamics of the pyramid center measured by 315 nm excitation for WT, W94F and W337F. All transients are similar.

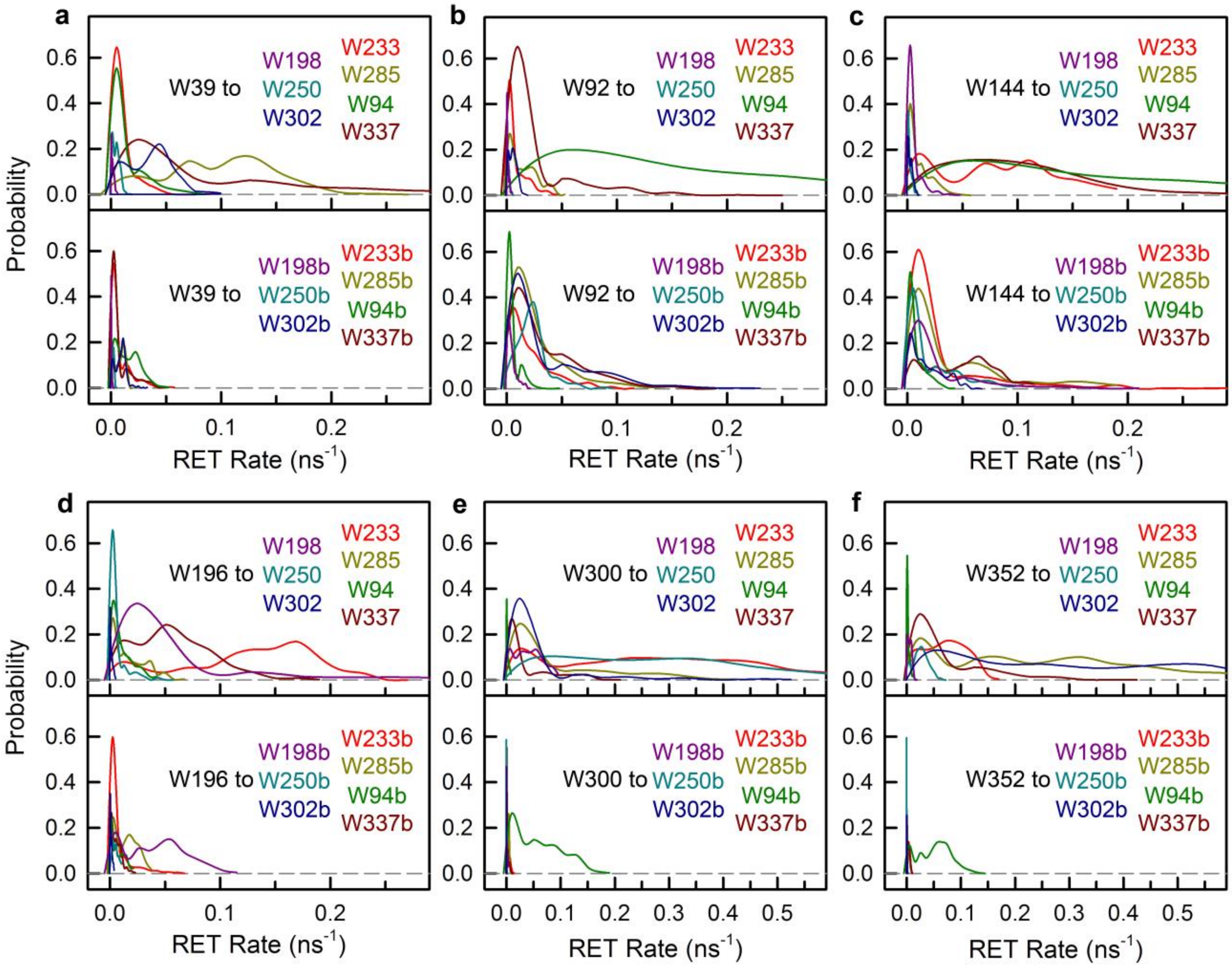


**Supplementary Figure 5 | RET rate distributions for all 84 $W_d$ to $W_p$/$W_c$ donor-acceptor pairs based on QM/MM calculations.** **a**, W39 as the donor. **b**, W92 as the donor. **c**, W144 as the donor. **d**, W196 as the donor. **e**, W300 as the donor. **f**, W352 as the donor. Top panels: energy transfer to tryptophan residues on the same subunit. Bottom panels: energy transfer to tryptophan residues on the other subunit.

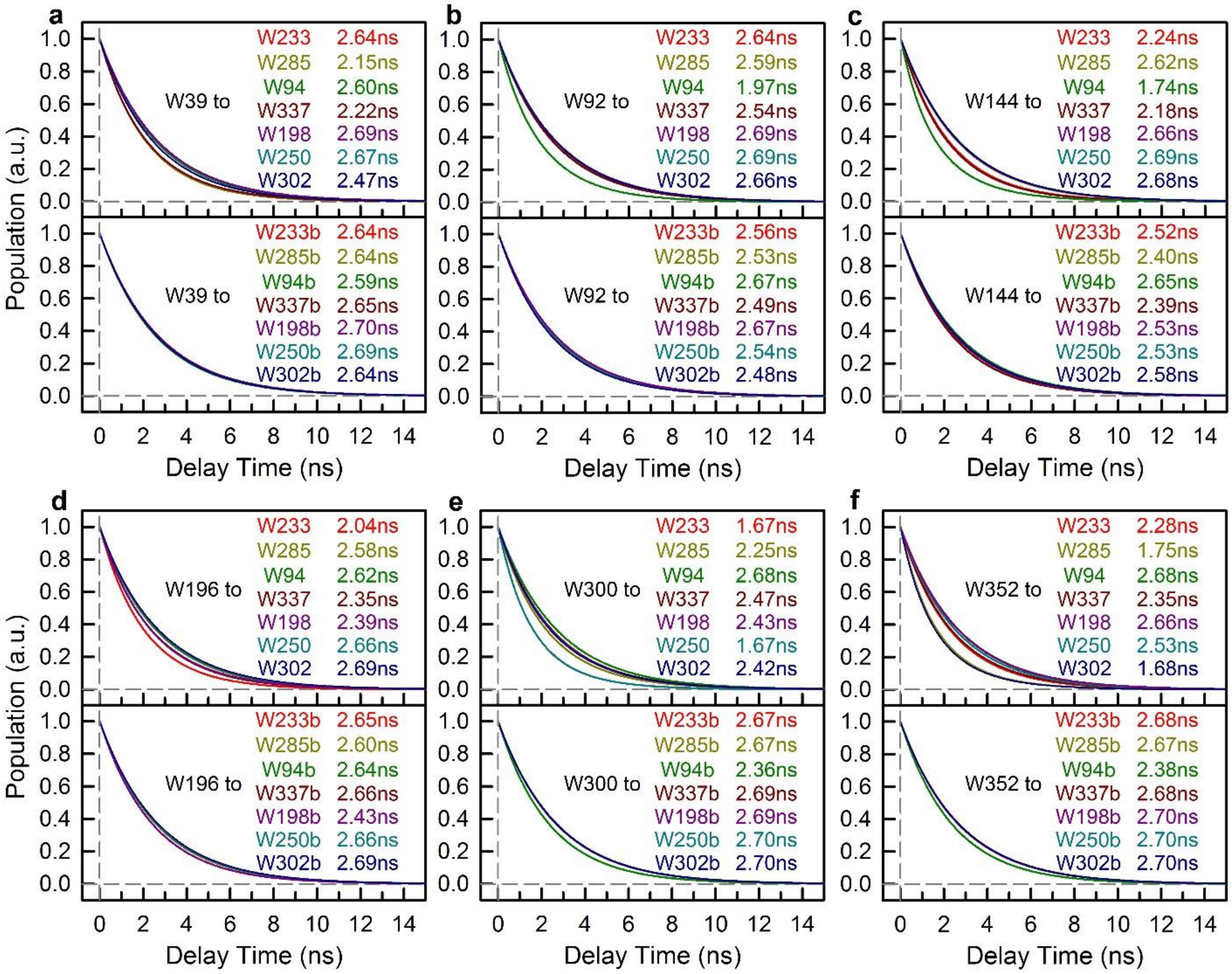


**Supplementary Figure 6 | Simulated excited state decay dynamics of $W_d$ in the presence of each individual energy acceptor.** **a**, W39 as the donor. **b**, W92 as the donor. **c**, W144 as the donor. **d**, W196 as the donor. **e**, W300 as the donor. **f**, W352 as the donor. Top panels: energy transfer to tryptophan residues on the same subunit. Bottom panels: energy transfer to tryptophan residues on the other subunit.

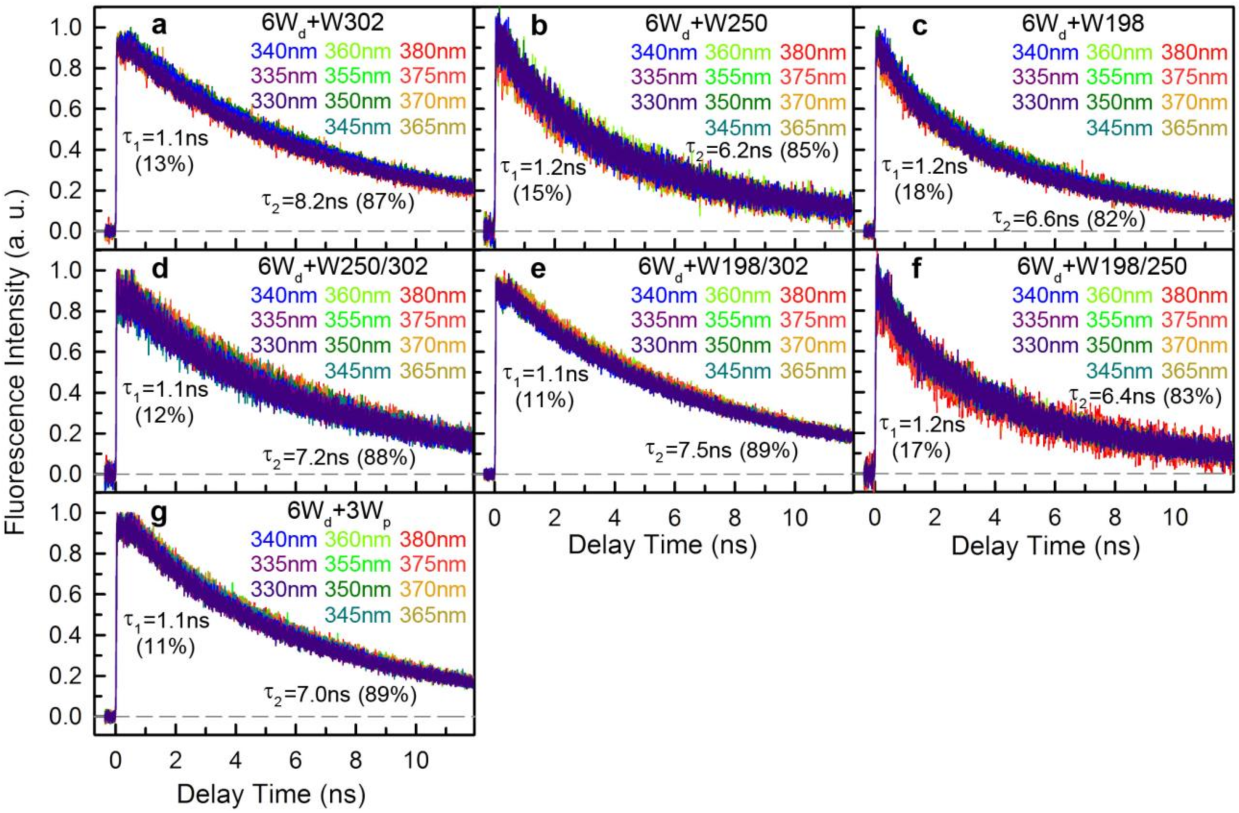


**Supplementary Figure 7 | Fluorescence decay transients ($\lambda_{ex}$=310 nm) from 330 nm to 380 nm for 8 mutants measured with picosecond resolved time correlated single photon counting (TCSPC).** **a**, 4$W_c$/198/250F (6$W_d$+W302); **b**, 4$W_c$/198/302F (6$W_d$+W250); **c**, 4$W_c$/250/302F (6$W_d$+W198); **d**, 4$W_c$/198F (6$W_d$+W250/302); **e**, 4$W_c$/250F (6$W_d$+W198/302); **f**, 4$W_c$/302F (6$W_d$+W198/250); **g**, 6$W_d$+3$W_p$. All these mutants contain 6$W_d$ and various $W_p$. For all mutants, transients at different wavelengths are very similar. Fitting results are shown for each mutant.

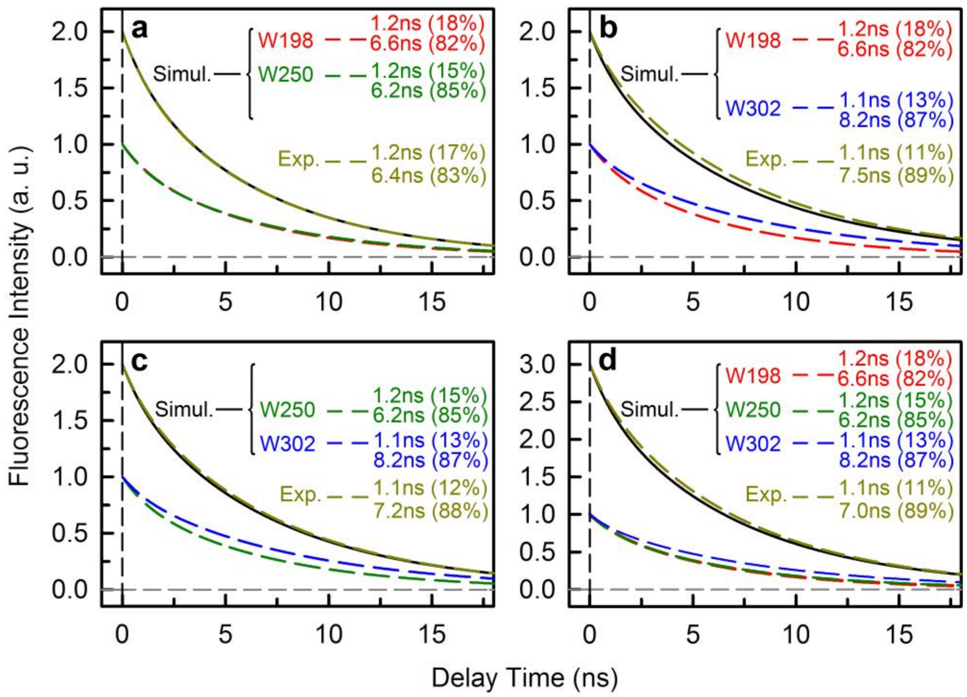


**Supplementary Figure 8 | Numerical simulations show that the $W_p$ lifetimes observed in $6W_d+2W_p$ mutants and $6W_d+3W_p$ mutants match the sum of decay transients of $6W_d+1W_p$.** The mutants: **a**, $4W_c$/198F ($6W_d$+W250/302); **b**, $4W_c$/250F ($6W_d$+W198/302); **c**, $4W_c$/302F ($6W_d$+W198/250); **d**, $6W_d+3W_p$. For each mutant, decay transients of individual $W_p$ (colored dashed lines, red-W198; green-W250; blue-W302) were simulated using double exponential decay model with the time constants and amplitude ratios (shown at the right side of the legend) measured from $6W_d+1W_p$ mutants, which add up a total decay transient (black solid line). The total transients agree with the simulated curves (orange solid lines) using time constants and ratios (shown in Supplementary Fig. 4) directly measured in "$6W_d+2W_p$" and "$6W_d+3W_p$" mutants.

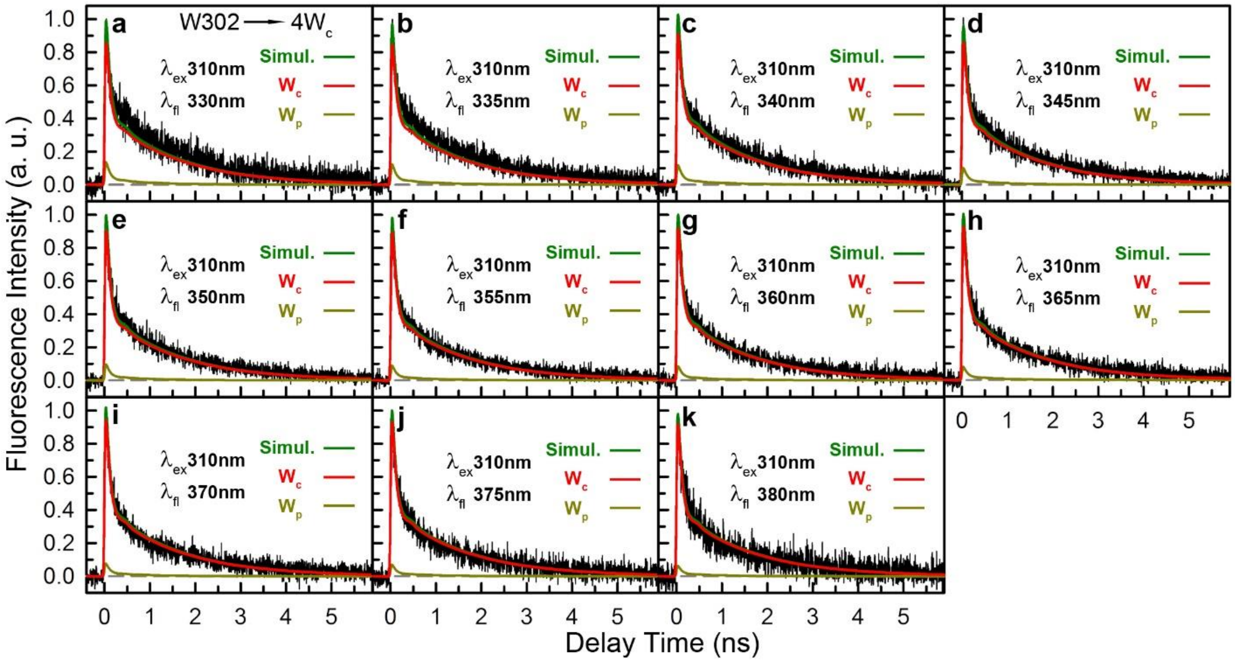


**Supplementary Figure 9 | Fluorescence decay transients ($\lambda_{ex}$=310 nm) for W198/250F measured with picosecond-resolved TCSPC agree with model simulations.** Transients were taken at various fluorescence wavelengths: **a**, 330 nm; **b**, 335 nm; **c**, 340 nm; **d**, 345 nm; **e**, 350 nm; **f**, 355 nm; **g**, 360 nm; **h**, 365 nm; **i**, 370 nm; **j**, 375 nm; and **k**, 380 nm. For each wavelength, the total simulated curve (green) and contributions from different tryptophan groups ($4W_c$, red; $3W_p$, dark yellow) are shown.

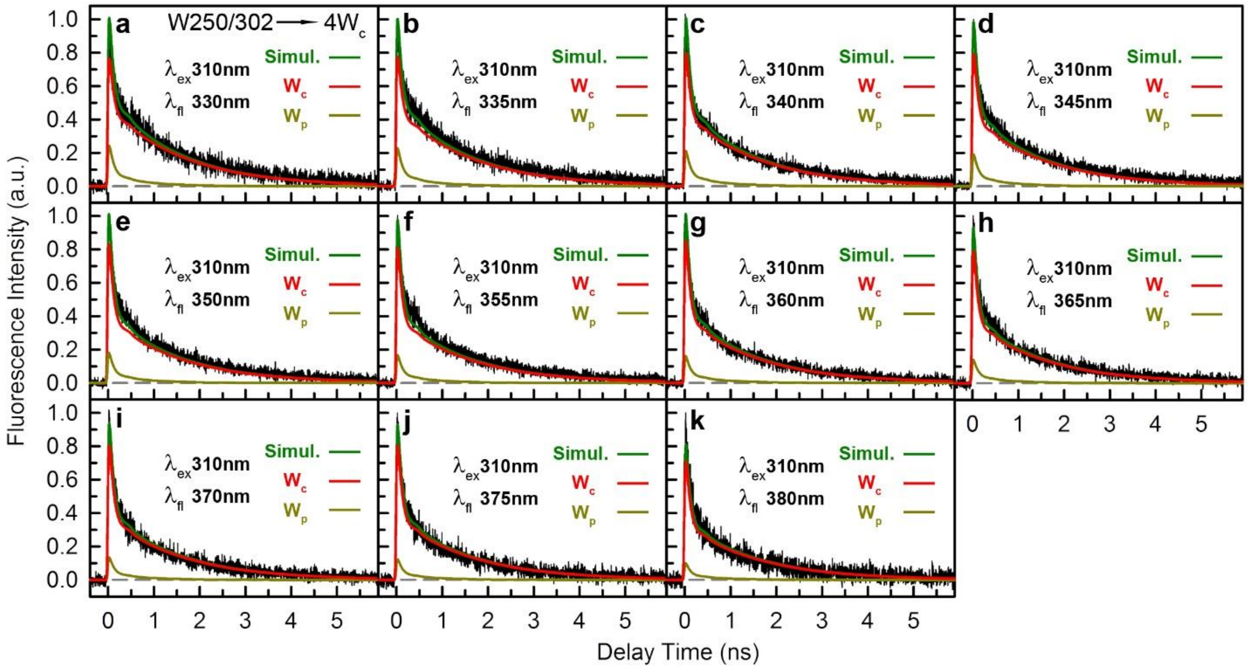


**Supplementary Figure 10 | Fluorescence decay transients ($\lambda_{ex}$=310 nm) for W198F measured with picosecond-resolved TCSPC agree with model simulations.** Transients were taken at various fluorescence wavelengths: **a**, 330 nm; **b**, 335 nm; **c**, 340 nm; **d**, 345 nm; **e**, 350 nm; **f**, 355 nm; **g**, 360 nm; **h**, 365 nm; **i**, 370 nm; **j**, 375 nm; and **k**, 380 nm. For each wavelength, the total simulated curve (green) and contributions from different tryptophan groups ($4W_c$, red; $3W_p$, dark yellow) are shown.

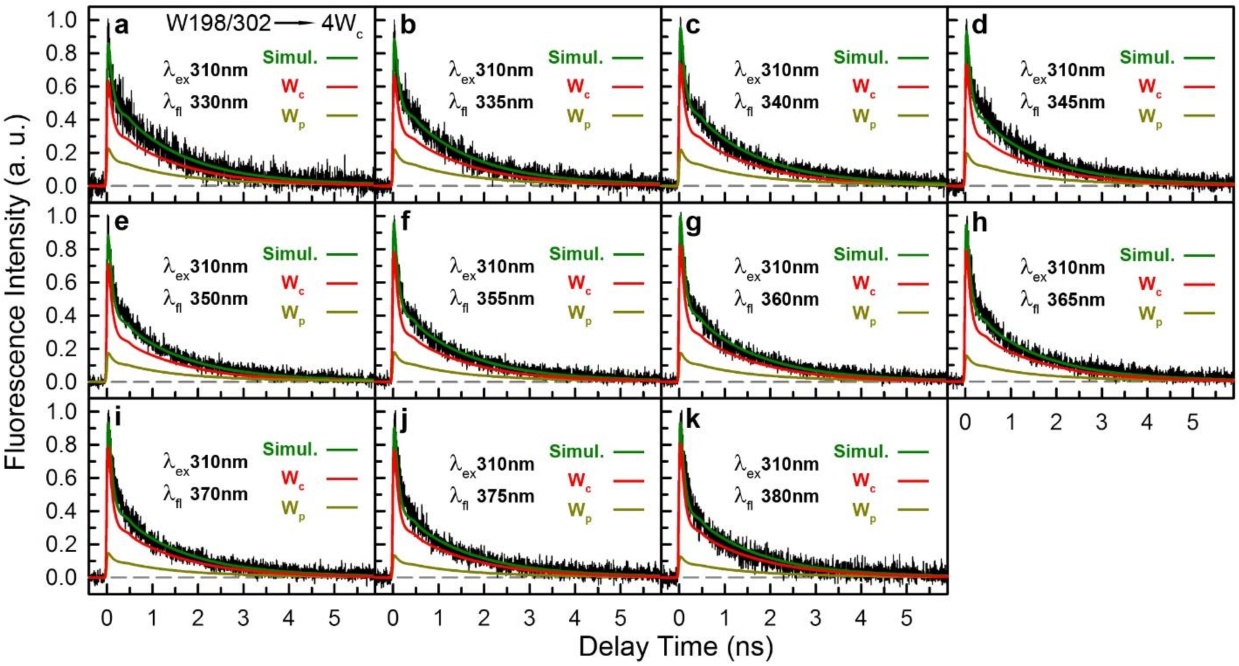


**Supplementary Figure 11 | Fluorescence decay transients ($\lambda_{ex}$=310 nm) for W250F measured with picosecond-resolved TCSPC agree with model simulations.** Transients were taken at various fluorescence wavelengths: **a**, 330 nm; **b**, 335 nm; **c**, 340 nm; **d**, 345 nm; **e**, 350 nm; **f**, 355 nm; **g**, 360 nm; **h**, 365 nm; **i**, 370 nm; **j**, 375 nm; and **k**, 380 nm. For each wavelength, the total simulated curve (green) and contributions from different tryptophan groups (4W$_c$, red; 3W$_p$, dark yellow) are shown.

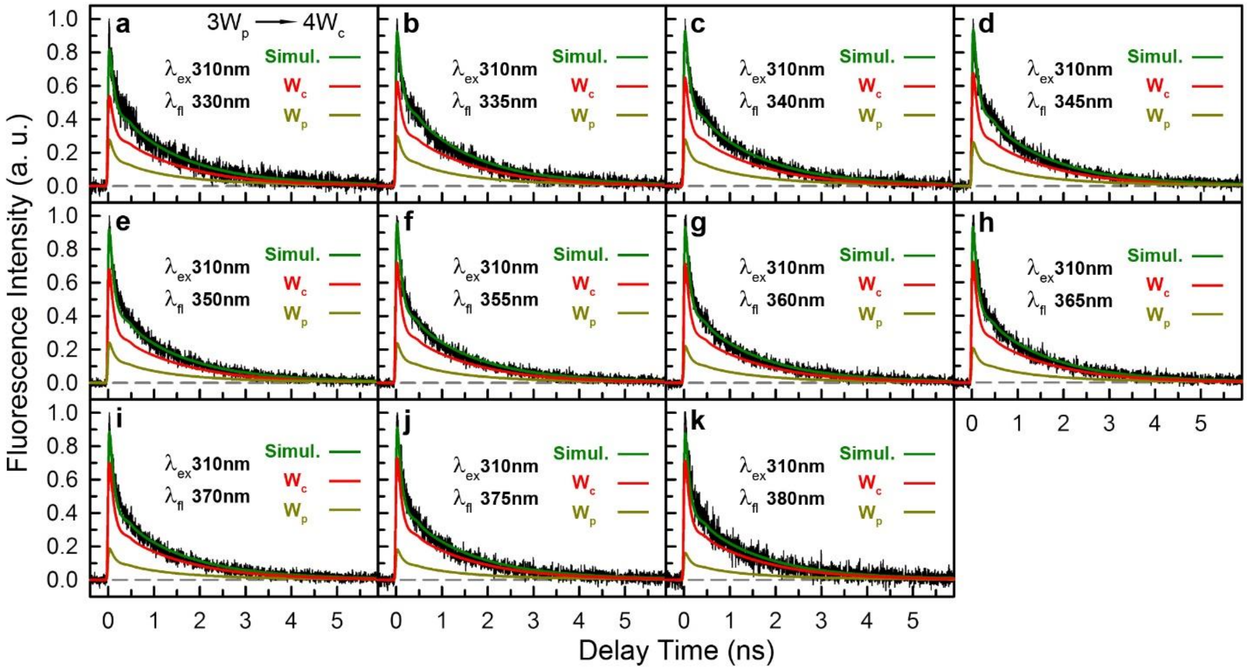


**Supplementary Figure 12 | Fluorescence decay transients ($\lambda_{ex}$=310 nm) for WT measured with picosecond-resolved TCSPC agree with model simulations.** Transients were taken at various fluorescence wavelengths: **a**, 330 nm; **b**, 335 nm; **c**, 340 nm; **d**, 345 nm; **e**, 350 nm; **f**, 355 nm; **g**, 360 nm; **h**, 365 nm; **i**, 370 nm; **j**, 375 nm; and **k**, 380 nm. For each wavelength, the total simulated curve (green) and contributions from different tryptophan groups ($4W_c$, red; $3W_p$, dark yellow) are shown.

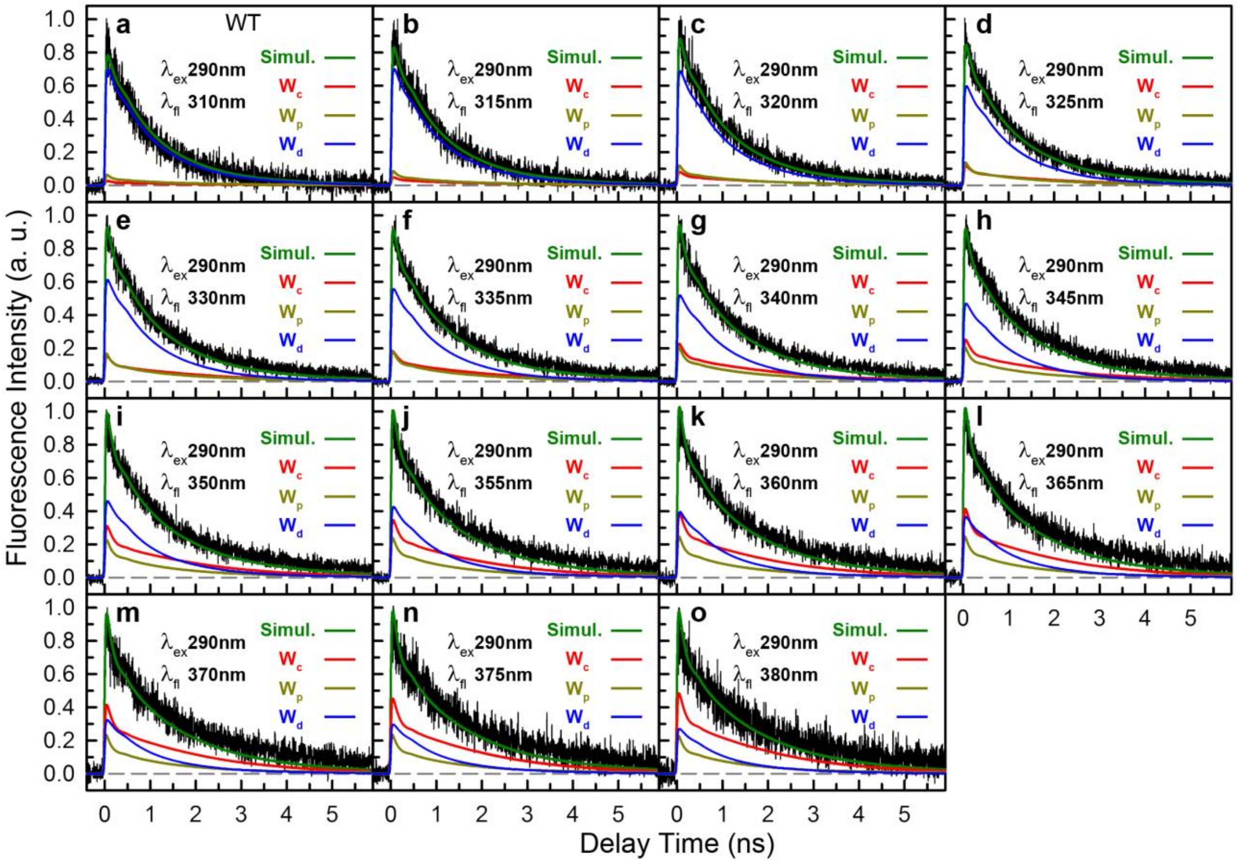


**Supplementary Figure 13 | Fluorescence decay transients ($\lambda_{ex}$=290 nm) for WT measured with picosecond-resolved TCSPC agree with model simulations.** Transients were taken at various fluorescence wavelengths: **a**, 310 nm; **b**, 315 nm; **c**, 320 nm; **d**, 325 nm; **e**, 330 nm; **f**, 335 nm; **g**, 340 nm; **h**, 345 nm; **i**, 350 nm; **j**, 355 nm; **k**, 360 nm; **l**, 365 nm; **m**, 370 nm; **n**, 375 nm; and **o**, 380 nm. For each wavelength, the total simulation curve (green) and contributions from different tryptophan groups (4$W_c$, red; 3$W_p$, dark yellow; 6$W_d$, blue) are shown. All simulation curves agree with experiments.

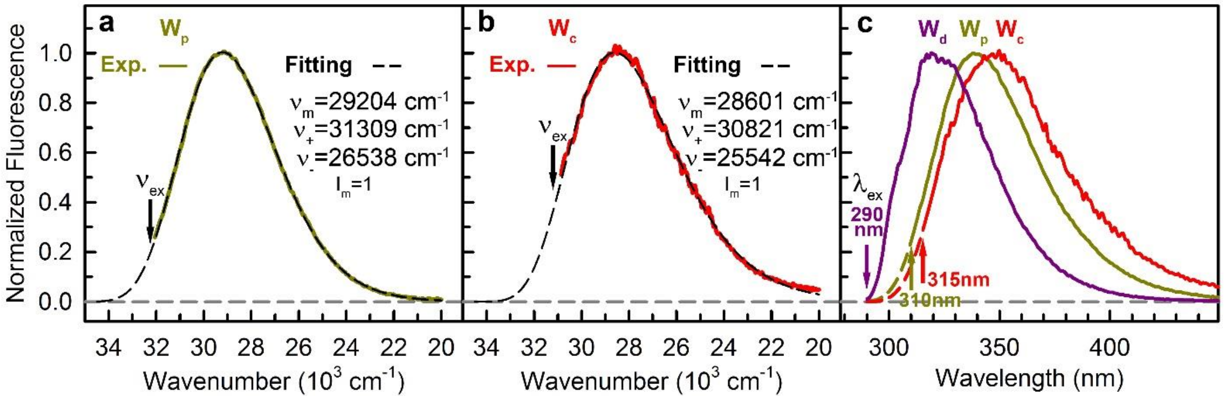


**Supplementary Figure 14 | Blue sides of the emission spectra extended by log-normal fitting.** **a**, $W_p$ emission spectra (in frequency domain) measured with 310 nm (32258 $cm^{-1}$) excitation is shown in dark yellow. The log-normal fitting curve is shown in a black dashed line. The four fitting parameters were labeled. **b**, $W_c$ emission spectra (in frequency domain) measured with 315 nm (31746 $cm^{-1}$) excitation is shown in red. The log-normal fitting curve is shown in a black dashed line. The four fitting parameters were labeled. **c**, Emission spectra of $W_d$, $W_p$ and $W_c$ in wavelength domain extended to 290 nm. The short-wavelength part from fitting are shown in dashed lines.

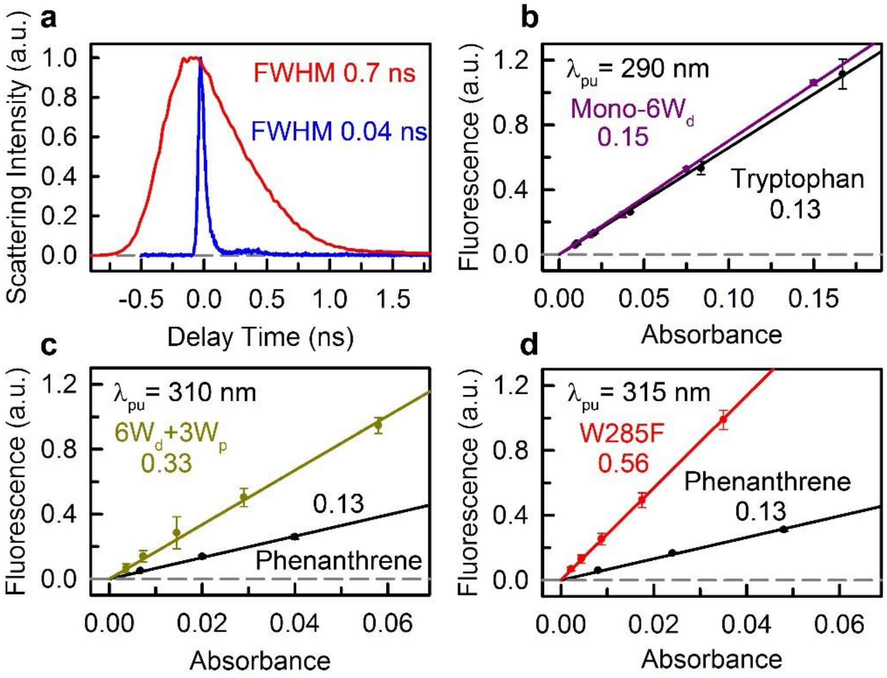


**Supplementary Figure 15 | Instrument response functions of TCSPC and fluorescence quantum yields of three tryptophan groups. a**, Instrument response functions for sub-nanosecond (red) and picosecond (blue) resolved TCSPC for data fitting and simulations. **b**, Fluorescence quantum yield of $6W_d$ as measured with mono-$6W_d$ (290 nm excitation) using tryptophan in aqueous solution as the standard. Radiative lifetime of $W_d$ is about 14 ns, as calculated with the fluorescence QY and lifetime. **c**, Fluorescence quantum yield of $3W_p$ as measured with $6W_d$+$3W_p$ (310 nm excitation) using phenanthrene in absolute ethanol as the standard. Radiative lifetime of $W_p$ is about 19 ns. **d**, Fluorescence quantum yield of pyramid center ($W_c$) without quenching as measured with W285F (315 nm excitation) using phenanthrene in absolute ethanol as the standard. The photoreaction was abolished by W285 mutation. Radiative lifetime of $W_c$ is about 20 ns.

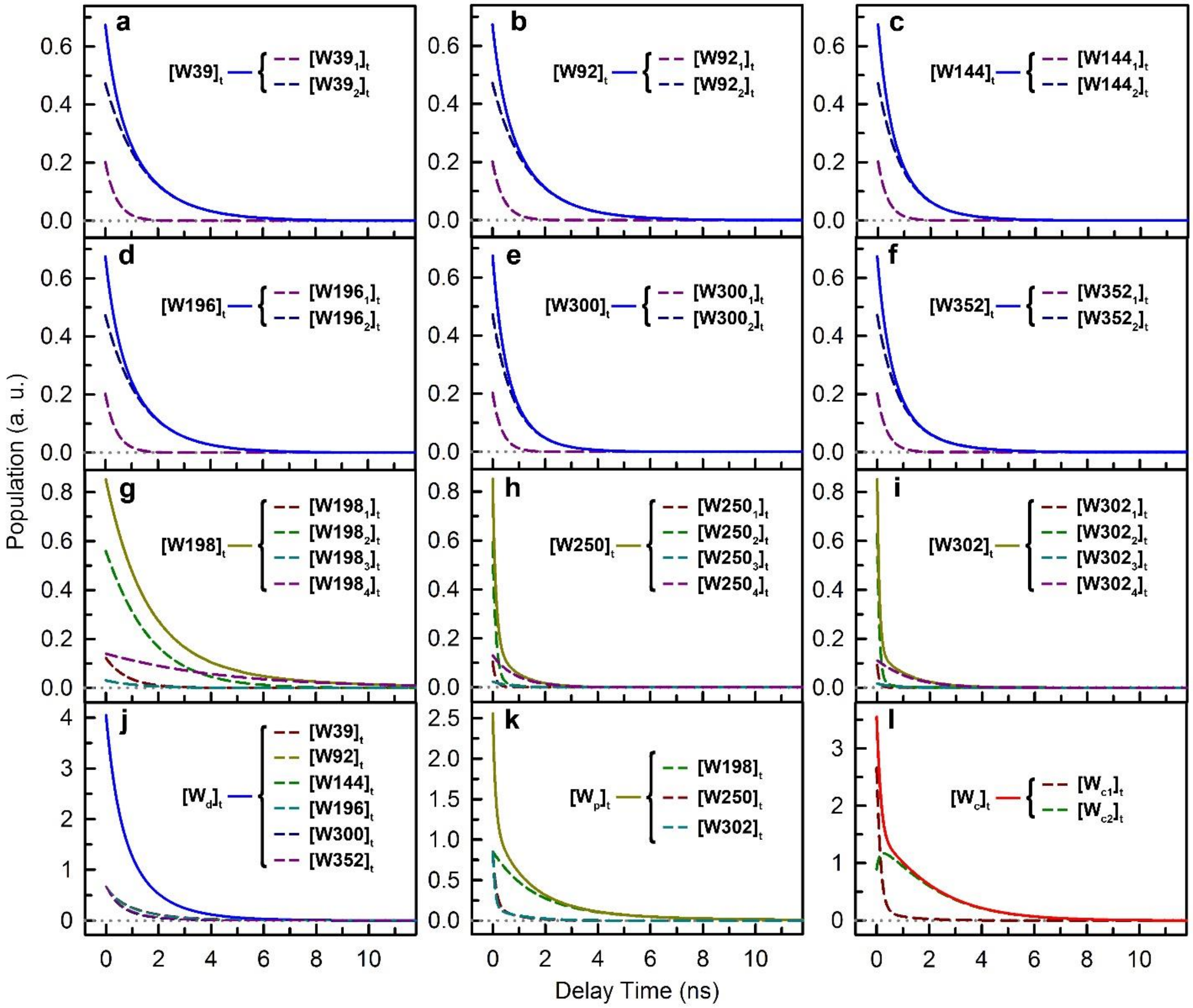


**Supplementary Figure 16 | Excited state population evolution of various W residues and W groups in WT at 290-nm excitation obtained by numerical simulations.** **a**, W39. **b**, W92. **c**, W144. **d**, W196. **e**, W300. **f**, W352. **g**, W198. **h**, W250. **i**, W302. **j**, 6$W_d$ group. **k**, 3$W_p$ group. **l**, 4$W_c$ group. The temporal evolution for each W residue or group (solid lines) is the sum of population evolution curves of subpopulations or individual W residue in the group (dashed lines).

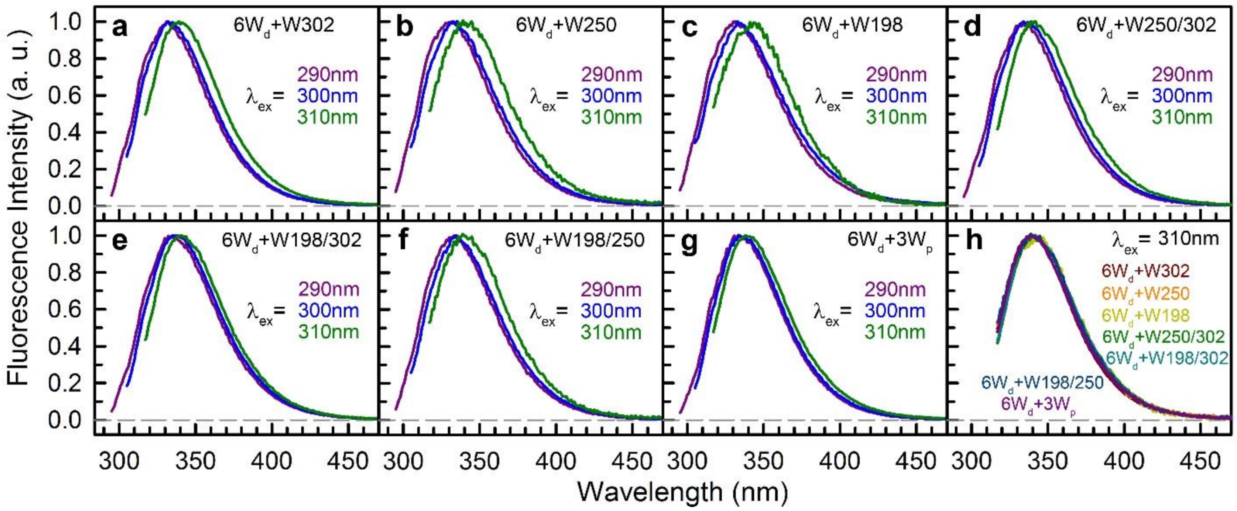


**Supplementary Figure 17 | Steady-state emission spectra measured with various excitation wavelengths for UVR8 mutants containing the 6 distal tryptophan residues and various $W_p$.** **a**, $4W_c$/198/250F ($6W_d$+W302); **b**, $4W_c$/198/302F ($6W_d$+W250); **c**, $4W_c$/250/302F ($6W_d$+W198); **d**, $4W_c$/198F ($6W_d$+W250/302); **e**, $4W_c$/250F ($6W_d$+W198/302); **f**, $4W_c$/302F ($6W_d$+W198/250); **g**, $6W_d$+$3W_p$; **h**, Steady-state emission spectra ($\lambda_{ex}$=310 nm) for all the mutants are nearly identical, indicating that the 3 peripheral tryptophan ($W_p$) have similar emissions with peaks at around 340 nm.

## Supplementary Tables

## Supplementary Table 1 | Mutant summary

| Mutant Type | Mutant | Abbreviated Name | State in Dark | UV Response |
|---|---|---|---|---|
| $6W_d$ | W94/233/285/337/198/250F/W302H[†] /R286A[*] | mono-$6W_d$ | Monomer | No |
| $6W_d+1W_p$ | W94/233/285/337/198/250F | $4W_c$/ 198/250F | Dimer | No |
| | W94/233/285/337/198/302F | $4W_c$/ 198/302F | Dimer | No |
| | W94/233/285/337/250/302F | $4W_c$/ 250/302F | Dimer | No |
| $6W_d+2W_p$ | W94/233/285/337/198F | $4W_c$/ 198F | Dimer | No |
| | W94/233/285/337/250F | $4W_c$/ 250F | Dimer | No |
| | W94/233/285/337/302F | $4W_c$/ 302F | Dimer | No |
| $6W_d+3W_p$ | W94/233/285/337F | $6W_d+3W_p$ | Dimer | No |
| $6W_d+4W_c$ | W198/250/302F | $6W_d+4W_c$ | Dimer | Yes |
| $6W_d+1W_p+$ $4W_c$ | W198/250F | W198/250F | Dimer | Yes |
| $6W_d+2W_p+$ $4W_c$ | W198F | W198F | Dimer | Yes |
| | W250F | W250F | Dimer | Yes |

[†]W94/233/285/337/198/250F/W302H/R286A mutant has higher protein yield than the phenylalanine mutant W94/233/285/337/198/250F/W302F/R286A

[*]Mutation of all peripheral tryptophan residues without knocking out R286 leads to no protein yield.

**Supplementary Table 2 | Overall RET efficiency of $6W_d$ in various systems.**

<table>
<tr><th>Mutant Type</th><th>Mutant</th><th>RET Scheme</th><th>$\tau_{DA2}$ (ns) (Exp.)*</th><th>$\tau_{Ave}$ (ns) (Exp.)†</th><th>E (Exp.)¶</th></tr>
<tr><td rowspan="3">$6W_d+1W_p$</td><td>$4W_c$/ 198/250F</td><td>$6W_d$ to W302</td><td>2.42±0.1</td><td>23±10</td><td>0.08±0.03</td></tr>
<tr><td>$4W_c$/ 198/302F</td><td>$6W_d$ to W250</td><td>2.37±0.1</td><td>19±6</td><td>0.10±0.03</td></tr>
<tr><td>$4W_c$/ 250/302F</td><td>$6W_d$ to W198</td><td>2.29±0.1</td><td>15±4</td><td>0.12±0.04</td></tr>
<tr><td rowspan="3">$6W_d+2W_p$</td><td>$4W_c$/198F</td><td>$6W_d$ to W250/302</td><td>2.28±0.1</td><td>15±4</td><td>0.12±0.04</td></tr>
<tr><td>$4W_c$/250F</td><td>$6W_d$ to W198/302</td><td>2.1±0.1</td><td>9.5±2</td><td>0.17±0.03</td></tr>
<tr><td>$4W_c$/302F</td><td>$6W_d$ to W198/250</td><td>2.16±0.1</td><td>10.6±2</td><td>0.16±0.04</td></tr>
<tr><td>$6W_d+3W_p$</td><td>$6W_d+3W_p$</td><td>$6W_d$ to $3W_p$</td><td>1.94±0.1</td><td>6.9±1.5</td><td>0.22±0.04</td></tr>
<tr><td>$6W_d+4W_c$</td><td>$6W_d+4W_c$</td><td>$6W_d$ to $4W_c$</td><td>1.50±0.1</td><td>3.4±0.5</td><td>0.35±0.03</td></tr>
<tr><td>WT</td><td>WT</td><td>$6W_d$ to $3W_p+4W_c$</td><td>1.20±0.1</td><td>2.2±0.3‡</td><td>0.45±0.03</td></tr>
</table>

*Experimental $\tau_{DA2}$ measured with nanosecond resolved TCSPC (Fig. 2b-f and Supplementary Fig. 2a-d). Fitting error is 0.1 ns.

†Obtained using experimental $\tau_{DA2}$ and 2.7 ns (1/(1/$\tau_{DA2}$-1/2.7). Errors were estimated based on 0.1 ns fitting errors of experimental $\tau_{DA2}$.

¶Overall RET efficiency from $6W_d$ estimated with equations 20 to 22, assuming the experimental $\tau_{Ave}$ in column 5 as the RET time for each individual $W_d$.

‡RET rate constant in WT matches the value (2.3 ns) calculated from 1/(1/6.9+1/3.4).

## Supplementary Table 3 | Calculated FRET time scales for all donor-acceptor pairs

| $\tau_{RET}$ (ns)[*] $W_d$ to $W_p/W_c$ | W39 | W92 | W144 | W196 | W300 | W352 |
|---|---|---|---|---|---|---|
| W94 | 72 (30) | 7.3 (5.4) | 4.9 (2050) | 85 (3510) | 344 (165) | 362 (100) |
| W233 | 111 (5390) | 115 (83) | 13 (6.0) | 8.3 (6.6) | 4.4 (4.7) | 15 (26) |
| W285 | 11 (6.3) | 67 (56) | 93 (106) | 58 (36) | 14 (16) | 4.9 (3.1) |
| W337 | 13 (161) | 43 (1680) | 11 (21) | 18 (84) | 29 (358) | 18 (294) |
| W94b | 64 (181) | 206 (100) | 137 (35) | 125 (65) | 18 (11) | 20 (12) |
| W233b | 121 (71) | 48 (89) | 39 (10) | 135 (60) | 275 (302) | 301 (165) |
| W285b | 127 (988) | 40 (13) | 22 (100) | 70 (44) | 278 (187) | 277 (236) |
| W337b | 135(409000) | 32 (13) | 21 (9.3) | 171 (147) | 558 (7740) | 329 (2400) |
| To Pyramid[†] | 4.2 (4.6) | 3.8 (2.6) | 2.0 (2.1) | 4.2 (3.9) | 2.5 (2.6) | 2.6 (2.2) |
| | | | | | | |
| W198 | 660 (1550) | 909(16100) | 194 (120) | 21 (9.3) | 24 (10) | 175 (139) |
| W250 | 212 (140) | 726 (366) | 483 (169) | 175 (160) | 4.4 (14) | 39 (28) |
| W302 | 29 (26) | 180 (112) | 362 (170) | 660 (588) | 24 (138) | 4.5 (7.3) |
| W198b | 2430(30300) | 218 (653) | 40 (314) | 25 (9.8) | 726 (328) | 2420(1440) |
| W250b | 605 (504) | 43 (39) | 40 (998) | 194 (296) | 7290(4170) | 8130(2100) |
| W302b | 115 (101) | 30 (97) | 59 (647) | 908 (109000) | 7320(1910) | 1460(745) |
| To peripheral[§] | 19 (17) | 15 (20) | 13 (39) | 9.8 (4.5) | 3.2 (5.6) | 3.9 (5.5) |
| $\tau_{total}$[**] | 3.6 (3.6) | 3.1 (2.3) | 1.7 (2.0) | 3.0 (2.1) | 1.4 (1.8) | 1.8 (1.6) |
| FRET Branching Ratios[¶] (Pyramid/Peripheral) | 3.7 | 8.0 | 18 | 1.1 | 2.2 | 2.6 |

| $\tau_{RET}$ (ns)[*] $W_p$ to $W_c$ | W198 | W250 | W302 |
|---|---|---|---|
| W94b | 14.0 | 2.1 | 5.7 |
| W233 | 3.2 | 0.12 | 44.4 |
| W285 | 45.7 | 0.63 | 0.05 |
| W337 | 12.0 | 518 | 2.3 |
| $\tau_{total}$[**] | 2.0 | 0.1 | 0.05 |

[*]$\tau_{RET}$ values for $W_d$ to $W_p/W_c$ were effective RET times defined using equation 15 based on 500 MD simulation snapshots. Values in brackets were calculated with supplementary equation 9 based on X-ray structure. $\tau_{RET}$ values for $W_p$ to $W_c$ were calculated with supplementary equation 9 based on X-ray structure.

[†]Total FRET time constants of the parallel 8 energy transfers to the 8 pyramid center W residues ($4W_c$ on both subunits).

[§]Total FRET time constants of the parallel 6 energy transfers to the 6 peripheral W residues ($3W_p$ on both subunits).

[**]Total energy transfer time scale for all parallel energy transfer pathways.

[¶]Obtained by dividing total peripheral FRET time constants by total pyramid FRET time constants.

**Supplementary Table 4 | Energy transfer efficiency of $W_p$ and $W_d$**

<table>
<tr><td></td><td colspan="2">W198</td><td colspan="2">W250</td><td colspan="2">W302</td></tr>
<tr><td>Fast energy transfer time constant ($\tau_{total1}$)</td><td colspan="2">1.8 ns</td><td colspan="2">0.12 ns</td><td colspan="2">0.08 ns</td></tr>
<tr><td>Slow energy transfer time constant ($\tau_{total2}$)</td><td colspan="2">12.3 ns</td><td colspan="2">1.0 ns</td><td colspan="2">1.1 ns</td></tr>
<tr><td>Population ratio of slow energy transfer ($R_{slow}$)</td><td colspan="2">0.20</td><td colspan="2">0.18</td><td colspan="2">0.15</td></tr>
<tr><td>First lifetime ($\tau_1$)</td><td colspan="2">1.2 ns (18%)</td><td colspan="2">1.2 ns (15%)</td><td colspan="2">1.1 ns (13%)</td></tr>
<tr><td>Second lifetime ($\tau_2$)</td><td colspan="2">6.6 ns (82%)</td><td colspan="2">6.2 ns (85%)</td><td colspan="2">8.2 ns (87%)</td></tr>
<tr><td>Energy transfer efficiency to $4W_c$*</td><td colspan="2">0.63</td><td colspan="2">0.94</td><td colspan="2">0.96</td></tr>
<tr><td>Overall efficiency $3W_p$ to $4W_c$</td><td colspan="6">0.84</td></tr>
<tr><td colspan="7"></td></tr>
<tr><td></td><td>W39</td><td>W92</td><td>W144</td><td>W196</td><td>W300</td><td>W352</td></tr>
<tr><td>Total RET efficiency (E)*</td><td>0.35</td><td>0.38</td><td>0.50</td><td>0.38</td><td>0.54</td><td>0.52</td></tr>
<tr><td>Overall efficiency $6W_d$ to $3W_p$+$4W_c$</td><td colspan="6">0.44</td></tr>
<tr><td>Efficiency of direct transfer to $4W_c$ ($E_{direct,4Wc}$)†</td><td>0.28</td><td>0.30</td><td>0.43</td><td>0.27</td><td>0.31</td><td>0.31</td></tr>
<tr><td>Efficiency to W198 ($E_{W198}$)§</td><td><0.01</td><td>0.01</td><td>0.03</td><td>0.10</td><td>0.03</td><td><0.01</td></tr>
<tr><td>Efficiency to W250 ($E_{W250}$)§</td><td>0.01</td><td>0.03</td><td>0.02</td><td>0.01</td><td>0.17</td><td>0.02</td></tr>
<tr><td>Efficiency to W302 ($E_{W302}$)§</td><td>0.05</td><td>0.04</td><td>0.02</td><td><0.01</td><td>0.03</td><td>0.18</td></tr>
<tr><td>Efficiency to $4W_c$ via $W_p$ ($E_{twostep}$)¶</td><td>0.06</td><td>0.07</td><td>0.05</td><td>0.08</td><td>0.21</td><td>0.20</td></tr>
<tr><td>Total RET efficiency to the pyramid center ($E_{4Wc}$)‡</td><td>0.34</td><td>0.37</td><td>0.49</td><td>0.35</td><td>0.52</td><td>0.51</td></tr>
<tr><td>Overall efficiency $6W_d$ to $4W_c$</td><td colspan="6">0.43</td></tr>
</table>

*The fraction of $W_d$ excited energy that is transferred to interfacial W residues.

†The fraction of $W_d$ excited energy that is directly transferred to the pyramid center.

§The fraction of $W_d$ excited energy that is transferred to each of the $3W_p$.

¶The fraction of $W_d$ excited energy that is transferred to the pyramid center via $3W_p$.

‡The fraction of $W_d$ excited energy that is transferred to the pyramid center, both directly and indirectly. The values are smaller than the total efficiency, meaning that a small fraction of energy dissipates in the two-step channels via $W_p$.

**Supplementary Table 5 | $A_d$, $A_p$ and $A_c$ values used in model simulations and excitation population percentage calculations.**

| $A_d$(310 nm) | $A_p$(310 nm) | $A_c$(310 nm) | $A_d$(290 nm) | $A_p$(290 nm) | $A_c$(290 nm) |
|---|---|---|---|---|---|
| 0 | 0.044 | 0.109 | 0.668 | 0.846 | 0.888 |

The values are direct readings of absorption curves in Fig. 1e. Initial excited populations of tryptophan residues are proportional to absorbance values, thus, we used the absorbance values as the initial populations in our model simulations. The same $A_d$ value was used for all the 6 different $W_d$. The same $A_p$ value was used for W198, W250 and W302. In mutants where certain $W_p$ were knocked out, the $A_p$ value for the corresponding $W_p$ was set to 0 in our simulations. For 290 nm excitation, excited state population distributions among three groups can be calculated with absorbance values: $W_d$, (0.668*6)/(0.668*6+0.846*3+0.888*4)=40%; $W_p$ (0.846*3)/(0.668*6+0.846*3+0.888*4)=25%; $W_c$, (0.888*4)/(0.668*6+0.846*3+0.888*4)=35%.

**Supplementary Table 6 | Fitting parameters for sub-nanosecond TCSPC transients of $4W_c$/198/250F ($6W_d$+W302) with the multiple-exponential decay model (equation 5).**

| Fluorescence Wavelength (nm) | Amplitude Percentage for Different Time Constants | | |
|---|---|---|---|
| | 0.5 ns | 2.43 ns | 8.21 ns |
| 305 | 49.8 | 47.6 | 2.6 |
| 310 | 45.2 | 51.0 | 3.8 |
| 315 | 38.7 | 55.3 | 6.0 |
| 320 | 34.3 | 57.0 | 8.7 |
| 325 | 30.4 | 57.9 | 11.7 |
| 330 | 25.5 | 59.3 | 15.3 |
| 335 | 23.0 | 57.8 | 19.2 |
| 340 | 19.6 | 57.3 | 23.1 |
| 345 | 17.1 | 55.6 | 27.4 |
| 350 | 13.0 | 55.1 | 31.9 |
| 355 | 10.2 | 53.2 | 36.6 |
| 360 | 8.1 | 51.2 | 40.6 |
| 365 | 8.3 | 47.7 | 44.1 |
| 370 | 0.6 | 48.4 | 51.0 |
| 375 | 3.0 | 44.3 | 52.7 |
| 380 | 0.2 | 43.0 | 56.8 |

**Supplementary Table 7 | Fitting parameters for sub-nanosecond TCSPC transients of $4W_c$/198/302F ($6W_d$+W250) with the multiple-exponential decay model (equation 5).**

| Fluorescence Wavelength (nm) | Amplitude Percentage for Different Time Constants | | |
|---|---|---|---|
| | 0.5 ns | 2.41 ns | 6.23 ns |
| 305 | 36.8 | 58.1 | 5.0 |
| 310 | 33.1 | 60.0 | 6.9 |
| 315 | 28.8 | 61.5 | 9.6 |
| 320 | 25.4 | 62.0 | 12.6 |
| 325 | 21.2 | 62.8 | 16.1 |
| 330 | 20.4 | 60.4 | 19.2 |
| 335 | 16.9 | 60.0 | 23.1 |
| 340 | 17.7 | 56.3 | 26.0 |
| 345 | 15.0 | 55.0 | 30.0 |
| 350 | 16.4 | 51.0 | 32.6 |
| 355 | 13.3 | 50.3 | 36.4 |
| 360 | 12.7 | 47.7 | 39.6 |
| 365 | 14.3 | 43.9 | 41.9 |
| 370 | 9.8 | 44.2 | 46.0 |
| 375 | 13.8 | 39.2 | 47.0 |
| 380 | 12.2 | 37.9 | 49.9 |

**Supplementary Table 8 | Fitting parameters for sub-nanosecond TCSPC transients of $4W_c$/250/302F ($6W_d$+W198) with the multiple-exponential decay model (equation 5).**

| Fluorescence Wavelength (nm) | Amplitude Percentage for Different Time Constants | | |
|---|---|---|---|
| | 0.5 ns | 2.18 ns | 6.58 ns |
| 305 | 33.8 | 60.9 | 5.3 |
| 310 | 32.1 | 60.9 | 7.0 |
| 315 | 28.7 | 62.0 | 9.3 |
| 320 | 26.7 | 61.2 | 12.1 |
| 325 | 25.6 | 59.1 | 15.3 |
| 330 | 21.0 | 60.2 | 18.8 |
| 335 | 18.8 | 58.6 | 22.6 |
| 340 | 17.1 | 56.3 | 26.6 |
| 345 | 17.4 | 52.8 | 29.8 |
| 350 | 14.2 | 51.2 | 34.6 |
| 355 | 13.0 | 48.5 | 38.5 |
| 360 | 12.7 | 45.3 | 42.1 |
| 365 | 10.3 | 44.0 | 45.7 |
| 370 | 11.7 | 39.5 | 48.7 |
| 375 | 9.6 | 38.4 | 51.9 |
| 380 | 7.7 | 36.8 | 55.5 |

**Supplementary Table 9 | Fitting parameters for sub-nanosecond TCSPC transients of $4W_c$/198F ($6W_d$+W250/302) with the multiple-exponential decay model (equation 5).**

| Fluorescence Wavelength (nm) | Amplitude Percentage for Different Time Constants | | |
|---|---|---|---|
| | 0.5 ns | 2.28 ns | 7.15 ns |
| 305 | 41.1 | 55.3 | 3.6 |
| 310 | 39.5 | 54.6 | 6.0 |
| 315 | 37.2 | 53.5 | 9.3 |
| 320 | 29.1 | 56.7 | 14.2 |
| 325 | 28.9 | 52.7 | 18.5 |
| 330 | 26.2 | 50.4 | 23.4 |
| 335 | 23.5 | 47.7 | 28.8 |
| 340 | 21.0 | 44.7 | 34.3 |
| 345 | 18.9 | 41.4 | 39.7 |
| 350 | 16.8 | 38.2 | 45.0 |
| 355 | 15.8 | 34.8 | 49.5 |
| 360 | 14.2 | 31.5 | 54.2 |
| 365 | 12.9 | 28.5 | 58.5 |
| 370 | 12.3 | 24.8 | 62.8 |
| 375 | 10.0 | 22.9 | 67.1 |
| 380 | 11.1 | 18.9 | 70.0 |

**Supplementary Table 10 | Fitting parameters for sub-nanosecond TCSPC transients of $4W_c$/250F ($6W_d$+W198/302) with the multiple-exponential decay model (equation 5).**

| Fluorescence Wavelength (nm) | Amplitude Percentage for Different Time Constants | | |
|---|---|---|---|
| | 0.5 ns | 2.10 ns | 7.54 ns |
| 305 | 42.5 | 53.3 | 4.2 |
| 310 | 38.1 | 55.1 | 6.8 |
| 315 | 35.0 | 54.1 | 10.9 |
| 320 | 29.1 | 55.3 | 15.7 |
| 325 | 27.4 | 51.6 | 21.0 |
| 330 | 24.6 | 48.6 | 26.8 |
| 335 | 22.3 | 45.2 | 32.5 |
| 340 | 19.7 | 41.5 | 38.8 |
| 345 | 17.2 | 37.5 | 45.3 |
| 350 | 15.2 | 33.8 | 50.9 |
| 355 | 14.7 | 29.4 | 55.9 |
| 360 | 12.9 | 25.2 | 61.8 |
| 365 | 11.0 | 21.8 | 67.2 |
| 370 | 10.0 | 17.4 | 72.6 |
| 375 | 9.8 | 14.6 | 75.6 |
| 380 | 11.3 | 9.5 | 79.2 |

**Supplementary Table 11 | Fitting parameters for sub-nanosecond TCSPC transients of 4W$_c$/302F (6W$_d$+W198/250) with the multiple-exponential decay model (equation 5).**

| Fluorescence Wavelength (nm) | Amplitude Percentage for Different Time Constants | | |
|---|---|---|---|
| | 0.5 ns | 2.15 ns | 6.36 ns |
| 305 | 39.2 | 55.4 | 5.4 |
| 310 | 35.4 | 56.2 | 8.4 |
| 315 | 32.7 | 56.1 | 11.1 |
| 320 | 27.4 | 57.4 | 15.2 |
| 325 | 23.7 | 56.7 | 19.6 |
| 330 | 21.5 | 54.8 | 23.7 |
| 335 | 18.0 | 54.4 | 27.6 |
| 340 | 15.4 | 52.6 | 32.1 |
| 345 | 10.6 | 52.4 | 37.0 |
| 350 | 11.3 | 48.8 | 39.9 |
| 355 | 9.6 | 46.8 | 43.6 |
| 360 | 6.6 | 45.5 | 47.8 |
| 365 | 7.7 | 41.8 | 50.5 |
| 370 | 5.4 | 40.6 | 54.0 |
| 375 | 2.5 | 39.8 | 57.6 |
| 380 | 2.0 | 38.0 | 60.0 |

**Supplementary Table 12 | Fitting parameters for sub-nanosecond TCSPC transients of $6W_d$+$3W_p$ with the multiple-exponential decay model (equation 5).**

| Fluorescence Wavelength (nm) | Amplitude Percentage for Different Time Constants | | |
|---|---|---|---|
| | 0.5 ns | 1.94 ns | 6.98 ns |
| 305 | 23.2 | 68.5 | 8.3 |
| 310 | 23.2 | 64.2 | 12.6 |
| 315 | 18.4 | 61.9 | 19.7 |
| 320 | 19.0 | 56.0 | 25.0 |
| 325 | 18.3 | 50.9 | 30.8 |
| 330 | 16.9 | 46.0 | 37.1 |
| 335 | 13.5 | 43.4 | 43.1 |
| 340 | 11.1 | 38.9 | 50.0 |
| 345 | 8.5 | 35.2 | 56.2 |
| 350 | 4.8 | 32.9 | 62.3 |
| 355 | 8.1 | 26.9 | 65.0 |
| 360 | 6.8 | 24.2 | 69.0 |
| 365 | 7.0 | 20.4 | 72.6 |
| 370 | 9.0 | 15.1 | 75.9 |
| 375 | 5.4 | 13.3 | 81.3 |
| 380 | 7.0 | 9.9 | 83.1 |

**Supplementary Table 13 | Fitting parameters for sub-nanosecond TCSPC transients of $6W_d$+$4W_c$ with the multiple-exponential decay model (equation 5).**

| Fluorescence Wavelength (nm) | Amplitude Percentage for Different Time Constants | | | |
|---|---|---|---|---|
| | 0.45 ns | 1.50 ns | 1.42 ns | 5.41 ns |
| 305 | 58.3 | 39.5 | 0.8 | 1.4 |
| 310 | 51.2 | 45.8 | 1.1 | 1.9 |
| 315 | 49.6 | 46.7 | 1.3 | 2.4 |
| 320 | 46.1 | 49.2 | 1.7 | 3.0 |
| 325 | 46.0 | 48.8 | 1.6 | 3.6 |
| 330 | 45.2 | 49.0 | 1.6 | 4.2 |
| 335 | 43.7 | 47.1 | 4.4 | 4.9 |
| 340 | 43.0 | 44.8 | 6.7 | 5.5 |
| 345 | 43.7 | 42.2 | 8.1 | 5.9 |
| 350 | 41.2 | 41.6 | 10.7 | 6.6 |
| 355 | 43.4 | 37.0 | 12.9 | 6.6 |
| 360 | 43.5 | 32.4 | 17.3 | 6.8 |
| 365 | 42.3 | 29.2 | 21.4 | 7.1 |
| 370 | 45.5 | 27.4 | 19.7 | 7.4 |
| 375 | 45.4 | 24.9 | 22.5 | 7.1 |
| 380 | 43.4 | 22.5 | 26.6 | 7.5 |

**Supplementary Table 14 | Fitting parameters for sub-nanosecond TCSPC transients of WT with multiple-the exponential decay model (equation 5).**

| Fluorescence Wavelength (nm) | Amplitude Percentage for Different Time Constants | | | |
|---|---|---|---|---|
| | 0.45 ns | 1.18 ns | 1.4 ns | 6.9 ns |
| 305 | 58.7 | 38.2 | 2.2 | 0.9 |
| 310 | 59.6 | 30.3 | 9.4 | 0.7 |
| 315 | 53.8 | 35.6 | 9.6 | 1.1 |
| 320 | 52.7 | 33.5 | 12.4 | 1.3 |
| 325 | 53.4 | 29.9 | 15.2 | 1.5 |
| 330 | 50.9 | 29.3 | 17.9 | 1.9 |
| 335 | 49.2 | 29.8 | 18.8 | 2.3 |
| 340 | 50.5 | 25.6 | 21.1 | 2.7 |
| 345 | 48.9 | 23.8 | 24.7 | 2.6 |
| 350 | 44.9 | 23.9 | 27.4 | 3.8 |
| 355 | 49.9 | 18.9 | 28.0 | 3.2 |
| 360 | 49.5 | 17.1 | 30.4 | 3.0 |
| 365 | 44.7 | 17.7 | 33.6 | 4.0 |
| 370 | 45.7 | 16.9 | 33.6 | 3.9 |
| 375 | 40.3 | 16.3 | 38.7 | 4.7 |
| 380 | 43.3 | 16.0 | 35.4 | 5.3 |

**Supplementary Table 15 | Distances and $\kappa^2$ between distal and interfacial tryptophan residues.**

| *R (Å)* | W39 | W92 | W144 | W196 | W300 | W352 |
|---|---|---|---|---|---|---|
| W94 | 20.5 | 14.1 | 14.9 | 21.8 | 28.3 | 26.0 |
| W198 | 29.7 | 26.8 | 20.1 | 13.6 | 20.7 | 27.0 |
| W233 | 21.7 | 21.1 | 18.9 | 17.2 | 17.1 | 19.7 |
| W250 | 27.4 | 28.8 | 25.6 | 19.8 | 14.3 | 21.3 |
| W285 | 19.4 | 21.6 | 22.3 | 21.7 | 16.0 | 16.4 |
| W302 | 21.1 | 26.8 | 28.8 | 27.1 | 14.0 | 14.1 |
| W337 | 16.7 | 17.4 | 18.6 | 20.0 | 17.3 | 16.2 |
| | | | | | | |
| W94b | 23.3 | 23.8 | 23.9 | 23.8 | 21.5 | 21.9 |
| W198b | 32.9 | 25.2 | 18.7 | 20.6 | 33.9 | 35.9 |
| W233b | 27.0 | 21.0 | 19.6 | 24.2 | 32.0 | 31.1 |
| W250b | 30.8 | 21.6 | 18.8 | 25.9 | 38.1 | 37.0 |
| W285b | 24.3 | 18.4 | 20.1 | 27.0 | 33.0 | 30.1 |
| W302b | 25.2 | 18.0 | 22.1 | 31.5 | 38.2 | 33.6 |
| W337b | 24.7 | 21.0 | 22.6 | 27.6 | 31.4 | 28.9 |
| | | | | | | |
| $\kappa^2$ | W39 | W92 | W144 | W196 | W300 | W352 |
| W94 | 0.46 | 0.27 | 0.00 | 0.01 | 0.58 | 0.57 |
| W198 | 0.16 | 0.01 | 0.20 | 0.25 | 2.71 | 1.01 |
| W233 | 0.00 | 0.20 | 1.42 | 0.73 | 0.98 | 0.42 |
| W250 | 1.09 | 0.56 | 0.61 | 0.14 | 0.22 | 1.17 |
| W285 | 1.58 | 0.34 | 0.22 | 0.55 | 0.20 | 1.15 |
| W302 | 1.25 | 1.20 | 1.20 | 0.24 | 0.02 | 0.40 |
| W337 | 0.02 | 0.00 | 0.37 | 0.14 | 0.01 | 0.01 |
| | | | | | | |
| W94b | 0.17 | 0.34 | 0.99 | 0.53 | 1.70 | 1.69 |
| W198b | 0.02 | 0.14 | 0.05 | 2.80 | 1.67 | 0.53 |
| W233b | 1.03 | 0.18 | 1.00 | 0.63 | 0.66 | 1.03 |
| W250b | 0.61 | 0.94 | 0.02 | 0.37 | 0.26 | 0.44 |
| W285b | 0.04 | 0.57 | 0.12 | 1.64 | 1.30 | 0.59 |
| W302b | 0.92 | 0.13 | 0.06 | 0.00 | 0.59 | 0.70 |
| W337b | 0.00 | 1.21 | 2.64 | 0.56 | 0.02 | 0.05 |

All results were based on X-ray structures[16,17]. We used the midpoint of the $C_{3a}$-$C_{7a}$ bond of indole ring as the center of the indole chromophores for distance calculations.

**Supplementary Table 16 | $\kappa^2/R^6$ between distal (6W$_d$) and interfacial tryptophan residues (3W$_p$ and 4W$_c$) for all donor-acceptor pairs.**

| $\kappa^2/R^6$ ($10^{-9}$Å$^{-6}$) | W39 | W92 | W144 | W196 | W300 | W352 |
|---|---|---|---|---|---|---|
| W94 | 6.28 | 34.50 | 0.09 | 0.05 | 1.13 | 1.87 |
| W198 | 0.23 | 0.02 | 3.01 | 38.90 | 34.70 | 2.60 |
| W233 | 0.03 | 2.26 | 31.20 | 28.40 | 39.60 | 7.19 |
| W250 | 2.58 | 0.99 | 2.14 | 2.26 | 25.80 | 12.70 |
| W285 | 29.50 | 3.35 | 1.77 | 5.21 | 12.00 | 59.80 |
| W302 | 14.00 | 3.22 | 2.13 | 0.61 | 2.62 | 49.40 |
| W337 | 1.16 | 0.11 | 9.12 | 2.22 | 0.52 | 0.64 |
| To A* | 53.76 | 44.40 | 49.50 | 77.70 | 116.00 | 134.00 |
| | | | | | | |
| W94b | 1.03 | 1.87 | 5.36 | 2.88 | 17.00 | 15.30 |
| W198b | 0.01 | 0.55 | 1.15 | 36.80 | 1.10 | 0.25 |
| W233b | 2.62 | 2.09 | 17.90 | 3.10 | 0.62 | 1.13 |
| W250b | 0.72 | 9.18 | 0.36 | 1.22 | 0.09 | 0.17 |
| W285b | 0.19 | 14.60 | 1.86 | 4.27 | 1.00 | 0.79 |
| W302b | 3.56 | 3.73 | 0.56 | 0.00 | 0.19 | 0.49 |
| W337b | 0.00 | 14.20 | 20.00 | 1.27 | 0.02 | 0.08 |
| To B† | 8.14 | 46.20 | 47.20 | 49.60 | 20.10 | 18.20 |

*Sum of the 7 $\kappa^2/R^6$ values to the interfacial W residues on the same subunit as the distal tryptophan donor.

†Sum of the 7 $\kappa^2/R^6$ values to the interfacial W residues on the other subunit.

**Supplementary Table 17 | Distances and $\kappa^2/R^6$ between $3W_p$ and $4W_c$.**

| *R (Å)* | W198 | W250 | W302 |
|---|---|---|---|
| W94b | 13.1 | 11.2 | 10.7 |
| W233 | 8.6 | 8.2 | 11.5 |
| W285 | 13.2 | 9.2 | 7.1 |
| W337 | 13.9 | 12.3 | 10.7 |
| | | | |
| W94 | 17.8 | 21.2 | 21.2 |
| W233b | 17.5 | 21.8 | 23.4 |
| W285b | 21.3 | 24.4 | 23.9 |
| W337b | 19.9 | 22.0 | 21.1 |
| | | | |
| $\kappa^2/R^6$ ($10^{-9}$Å$^{-6}$) | W198 | W250 | W302 |
| W94b | 70.5 | 478 | 172 |
| W233 | 310 | 8250 | 22.2 |
| W285 | 21.6 | 1560 | 19000 |
| W337 | 82.3 | 19.1 | 421 |
| To the closer pyramid* | 485 | 10000 | 20000 |
| | | | |
| W94 | 2.0 | 1.6 | 1.0 |
| W233b | 15.1 | 1.1 | 0.5 |
| W285b | 18.7 | 6.5 | 10.9 |
| W337b | 5.6 | 30 | 33.7 |
| To the further pyramid† | 41 | 39 | 46 |

Results were based on X-ray structures[16,17]. We used the midpoint of the $C_{3a}$-$C_{7a}$ bond of indole ring as the center of the indole chromophores for distance calculations.

*Sum of the 4 $\kappa^2/R^6$ values to the closer $4W_c$.

†Sum of the 4 $\kappa^2/R^6$ values to the further $4W_c$.

**Supplementary Table 18 | Lifetimes and amplitude ratios used in model simulations and energy transfer efficiency calculations.**

| Description | Symbol | Value |
|---|---|---|
| The first lifetime of $W_d$ | $\tau_1$ | 0.5 ns |
| The second lifetime of $W_d$ | $\tau_2$ | 2.7 ns |
| Amplitude ratio of the second lifetime of $W_d$ | $R_{D2}$ | 0.71 |
| The first lifetime of W198 | $\tau_{1,W198}$ | 1.2 ns |
| The second lifetime of W198 | $\tau_{2,W198}$ | 6.6 ns |
| Amplitude ratio of the second lifetime of W198 | $R_{2,W198}$ | 0.82 |
| Fast energy transfer time constant of W198 | $\tau_{total1,W198}$ | 1.8 ns |
| Slow energy transfer time constant of W198 | $\tau_{total2,W198}$ | 12.3 ns |
| Amplitude ratio of slow energy transfer in W198 | $R_{slow,W198}$ | 0.20 |
| The first lifetime of W250 | $\tau_{1,W250}$ | 1.2 ns |
| The second lifetime of W250 | $\tau_{2,W250}$ | 6.2 ns |
| Amplitude ratio of the second lifetime of W250 | $R_{2,W250}$ | 0.85 |
| Fast energy transfer time constant of W250 | $\tau_{total1,W250}$ | 0.12 ns |
| Slow energy transfer time constant of W250 | $\tau_{total2,W250}$ | 1.0 ns |
| Amplitude ratio of slow energy transfer in W250 | $R_{slow,W250}$ | 0.18 |
| The first lifetime of W302 | $\tau_{1,W302}$ | 1.1 ns |
| The second lifetime of W302 | $\tau_{2,W302}$ | 8.2 ns |
| Amplitude ratio of the second lifetime of W302 | $R_{2,W302}$ | 0.87 |
| Fast energy transfer time constant of W302 | $\tau_{total1,W302}$ | 0.08 ns |
| Slow energy transfer time constant of W302 | $\tau_{total2,W302}$ | 1.1 ns |
| Amplitude ratio of slow energy transfer in W302 | $R_{slow,W302}$ | 0.15 |
| The first fluorescence decay time of the pyramid center | $\tau_{c1}$ | 0.08 ns |
| The second fluorescence decay time of the pyramid center | $\tau_{c2}$ | 1.4 ns |
| Amplitude ratio of the 0.08 ns component | $R_{c1}$ | 0.75 |

**Supplementary Table 19 | Values of $FL_d$, $FL_p$ and $FL_c$ at various wavelengths for model simulations.**

| Wavelength (nm) | $FL_d$ (a.u.) | $FL_p$ (a.u.) | $FL_c$ (a.u.) |
|---|---|---|---|
| 310 | 1.46 | 0.247 | 0.068 |
| 315 | 1.84 | 0.421 | 0.150 |
| 320 | 1.89 | 0.612 | 0.268 |
| 325 | 1.83 | 0.786 | 0.433 |
| 330 | 1.79 | 0.916 | 0.541 |
| 335 | 1.60 | 0.987 | 0.636 |
| 340 | 1.35 | 1 | 0.72 |
| 345 | 1.17 | 0.963 | 0.772 |
| 350 | 0.969 | 0.891 | 0.790 |
| 355 | 0.768 | 0.798 | 0.755 |
| 360 | 0.601 | 0.695 | 0.711 |
| 365 | 0.477 | 0.593 | 0.650 |
| 370 | 0.372 | 0.497 | 0.580 |
| 375 | 0.279 | 0.411 | 0.518 |
| 380 | 0.216 | 0.336 | 0.469 |

$FL_d$, $FL_p$ and $FL_c$ are the fluorescence intensity per unit population per unit time for excited $W_d$, $W_p$ and $W_c$, respectively. Ratios of $FL_d$, $FL_p$ and $FL_c$ at various wavelengths give the weights of 3 tryptophan groups in total simulated signals (Supplementary equations 15, 16 and 17). For each group, plots of FL numbers versus wavelengths have the same shape as the emission spectrum of the tryptophan group shown in Fig. 1e. Their wavelength integrals were inversely proportional to the radiative lifetimes (inverse of radiative rates) of 3 W groups, which were estimated based on fluorescence quantum yields and lifetimes (Supplementary Fig. 15).

## References

1. Andrews, D. L. & Demidov, A. A. Resonance Energy Transfer (Wiley, Chichester, England, 1999).
